# Motor cortico-nigral and cortico-entopeduncular information transmission and its modulation by buspirone in control and after dopaminergic denervation

**DOI:** 10.3389/fphar.2022.953652

**Published:** 2022-08-30

**Authors:** Sergio Vegas-Suárez, Teresa Morera-Herreras, Catalina Requejo, José Vicente Lafuente, Rosario Moratalla, Cristina Miguélez, Luisa Ugedo

**Affiliations:** ^1^ Department of Pharmacology, Faculty of Medicine and Nursing, University of the Basque Country (UPV/EHU), Leioa, Spain; ^2^ Autonomic and Movement Disorders Unit, Neurodegenerative Diseases, Biocruces Health Research Institute, Barakaldo, Spain; ^3^ Cajal Institute, Spanish National Research Council (CSIC), Madrid, Spain; ^4^ Network Center for Biomedical Research in Neurodegenerative Diseases (CIBERNED), Carlos III Institute of Health (ISCIII), Madrid, Spain; ^5^ LaNCE, Department of Neuroscience, University of the Basque Country (UPV/EHU), Leioa, Spain

**Keywords:** 5-HT_1A_, electrophysiology, motor cortex, Parkinson’s disease, entopeduncular nucleus, substantia nigra pars reticulata, buspirone, WAY 100635

## Abstract

Cortical information is transferred to the *substantia nigra pars reticulata* (SNr) and the entopeduncular nucleus (EP), the output structures of the basal ganglia (BG), through three different pathways: the hyperdirect trans-subthalamic and the direct and indirect trans-striatal pathways. The nigrostriatal dopamine (DA) and the activation of 5-HT_1A_ receptors, distributed all along the BG, may modulate cortical information transmission. We aimed to investigate the effect of buspirone (5-HT_1A_ receptor partial agonist) and WAY-100635 (5-HT_1A_ receptor antagonist) on cortico-nigral and cortico-entopeduncular transmission in normal and DA loss conditions. Herein, simultaneous electrical stimulation of the motor cortex and single-unit extracellular recordings of SNr or EP neurons were conducted in urethane-anesthetized sham and 6-hydroxydopamine (6-OHDA)-lesioned rats before and after drug administrations. Motor cortex stimulation evoked monophasic, biphasic, or triphasic responses, combination of an early excitation, an inhibition, and a late excitation in both the SNr and EP, while an altered pattern of evoked response was observed in the SNr after 6-OHDA lesion. Systemic buspirone potentiated the direct cortico-SNr and cortico-EP transmission in sham animals since increased duration of the inhibitory response was observed. In DA denervated animals, buspirone administration enhanced early excitation amplitude in the cortico-SNr transmission. In both cases, the observed effects were mediated via a 5-HT_1A_-dependent mechanism as WAY-100635 administration blocked buspirone’s effect. These findings suggest that in control condition, buspirone potentiates direct pathway transmission and DA loss modulates responses related to the hyperdirect pathway. Overall, the results may contribute to understanding the role of 5-HT_1A_ receptors and DA in motor cortico-BG circuitry functionality.

## 1 Introduction

The basal ganglia (BG) are a group of subcortical nuclei that play an important role in the control of motor behavior, emotion, and cognition (Lanciego et al., 2012; Leisman et al., 2014). Thus, functional alterations in the BG are relevant in the etiopathology of movement disorders such as Parkinson’s disease (PD), a neurodegenerative disorder characterized by motor symptoms and dopaminergic (DA) neuronal loss (Kish et al., 1988). The BG nuclei, striatum (caudate and putamen), subthalamic nucleus (STN), internal segment of the *globus pallidus* (GPi) or entopeduncular nucleus (EP in rodents), and external segment of the *globus pallidus* (GPe), *substantia nigra pars reticulata* (SNr), and *substantia nigra pars compacta* (SNc) form a highly organized network connecting the cerebral cortex to the thalamus. All of them shape the BG-thalamo-cortical loop (Alexander et al., 1986). Motor cortical areas project to the striatum and the STN, while the final stage of the system arises from the GPi/EP and SNr, named the output nuclei (Alexander and Crutcher, 1990). These latter ones receive cortical information through three different pathways: 1) the hyperdirect or cortico-STN-EP and SNr pathways, 2) the direct cortico-striato-EP and SNr pathways, and 3) the indirect cortico-striato-GPe-STN-EP and SNr pathways (Nambu et al., 2000). DA neurons located in the SNc elicit an important control on the BG output nuclei directly and indirectly through the BG that cortico-BG pathways elicit on the SNr and EP (DeLong, 1990; Wahyu et al., 2021), leading to neuronal activity alteration (Galvan et al., 2015).

In the last decades, the need for new therapeutical compounds in PD have been focused on non-dopaminergic systems, as the serotonergic (5-HT) one, with special emphasis in 5-HT1A receptor mediators. The 5-HT neurons from the dorsal raphe nucleus (DRN) innervate almost all brain areas including the BG nuclei (Huang et al., 2019). DA loss is also associated with alterations in 5-HT neurotransmission coming from the raphe nuclei to the BG nuclei (Di Matteo et al., 2008) causing, among others, changes in the response mediated by 5-HT_1A_ receptor activation on cortical pyramidal neurons (Wang et al., 2009), STN (Sagarduy et al., 2016), SNr, and EP (Vegas-Suárez et al., 2020; Vegas-Suárez et al., 2021). The modulatory action of 5-HT_1A_ receptors on cortico-BG transmission might be changed by DA loss, as they are expressed throughout the whole BG (Lanfumey and Hamon, 2000). With all, studies evaluating 5-HT_1A_ receptors in different regions report variable results. In hemiparkinsonian rats, we have observed a slight but significant reduction in 5-HT_1A_ receptor immunoreactivity in the striatum and GPe after DA loss (Vegas-Suárez et al., 2021), in line with a micro-PET study (Vidal et al., 2021). The use of other techniques such as autoradiography and *in situ* hybridization for 5-HT_1A_ studies failed to support those results in the striatum (Radja et al., 1993; Numan et al., 1995). In MPTP-injected monkeys, increased 5-HT_1A_ receptor immunoreactivity was observed in the striatum, while decreased immunoreactivity was found in the DRN or motor and premotor cortical areas (Frechilla et al., 2001).

In the present study, we investigated the effect of 5-HT_1A_ receptor drugs on motor cortico-nigral and cortico-entopeduncular transmission in control and after DA loss conditions. We chose to study the effect of buspirone, a 5-HT_1A_ receptor agonist, as it has been proved to exert anxiolytic and antidyskinetic effects in parkinsonian animals and PD patients (Dekundy et al., 2007; Eskow et al., 2007; Aristieta et al., 2012; Politis et al., 2014) and anxiolytic properties in PD (Schneider et al., 2021). In this sense, several clinical trials have been published or are being carried out in humans. Efficacy of buspirone as antidyskinetic is being tested in four placebo-controlled randomized double-blind clinical trials, one phase III (NCT02617017), two phase II with co-administration of the 5- HT1B/1D agonist zolmitriptan (NCT03956979 and NCT04377945), and one phase I as co-therapy with amantadine (NCT02589340). In addition, a recent clinical trial (NCT02803749) has suggested that buspirone presented tolerability and some efficacy in the treatment of anxiety in some patients with PD (Schneider et al., 2021). Despite these beneficial therapeutic indications, the effect of buspirone on motor performance is not clear. Some preclinical studies support that it could have a beneficial effect on motor performance (Siemiatkowski et al., 2000; Nayebi et al., 2010; Nayebi and Sheidaei, 2010; Lindenbach et al., 2015), but others, including clinical and preclinical studies, report worsening of motor function when administered to parkinsonian animals or patients with PD (Dekundy et al., 2007; Lindenbach et al., 2015; Rissardo and Caprara, 2020). For all these reasons, it is interesting to understand how buspirone impacts information transmission along the BG in control and parkinsonian animals. Our previous studies showed that buspirone reduced burst activity of the output nuclei after DA loss. Present findings show that cortico-BG transmission is modified by DA loss, mainly affecting evoked responses related to the hyperdirect pathway in the SNr, while buspirone modulates evoked responses related to the direct pathway in both nuclei only in control conditions.

## 2 Materials and methods

### 2.1 Animals

A total of 94 male Sprague Dawley rats (SGIker facilities, UPV/EHU) weighing 150–175 g were randomly divided into two different experimental groups: sham (*n* = 49) and 6-hydroxydopamine (6-OHDA)-lesioned rats (*n* = 45). Animals were housed in groups of at least four animals under standard laboratory conditions (22 ± 1°C, 55 ± 5% relative humidity, and a 12:12 h light/dark cycle) with *ad libitum* access to food and water. The experimental protocol was approved by the Local Ethical Committee for Animal Research of the UPV/EHU (ref. CEEA/M20/2016/176) following the European (2010/63/UE) and Spanish (RD 53/2013) regulations for the care and use of laboratory animals. Despite being aware of the importance of gender inclusion in preclinical research studies, we only used male rats even though some differences could be found between male and female 6-OHDA-lesioned rats (Sagarduy et al., 2016). Every effort was made to minimize animal suffering and to use the minimum number of animals per group and experiment.

### 2.2 Drugs

6-OHDA hydrochloride, benserazide hydrochloride, desipramine hydrochloride, buspirone hydrochloride, and urethane were obtained from Sigma-Merck; isoflurane anesthetic was obtained from Esteve; and WAY-100365 maleate and pargyline hydrochloride were obtained from Tocris Bioscience. In each session, desipramine, pargyline, buspirone, and WAY-100365 were dissolved in 0.9% saline. 6-OHDA was prepared in Milli-Q water containing 0.02% ascorbic acid. Urethane was prepared in Milli-Q water.

### 2.3 6-Hydroxydopamine lesion

6-OHDA lesions were performed as described by Aristieta et al. (2016) and Aristieta et al. (2019). Thirty minutes before surgery, the rats were pre-treated with desipramine (25 mg/kg, i.p.) and pargyline (50 mg/kg, i.p.) to prevent damage to the noradrenergic system or to increase the selectivity and efficacy of toxin, respectively. Next, the rats were deeply anesthetized using isoflurane (4% for induction and 1.5–2.0% for maintenance) and placed in a stereotaxic frame (David Kopf^®^ Instruments). 6-OHDA (3.5 μg/μL, in 0.02% ascorbic acid) or vehicle was injected using a 10-μL Hamilton syringe at a rate of 1 μL/min in the coordinates of the right medial forebrain bundle: 2.5 μL at the anteroposterior (AP) (−4.4 mm), mediolateral (ML) (+1.2 mm), and dorsoventral (DV) (–7.8 mm) relative to bregma (Paxinos and Watson, 2007) with a toothbar set at −2.4 and 2 μL at AP (−4.0 mm), ML (+0.8 mm), and DV (−8 mm) with a toothbar at +3.4.

### 2.4 Cylinder test

Three weeks after the 6-OHDA lesion, forelimb asymmetry was evaluated (Vegas-Suárez et al., 2020; Vegas-Suárez et al., 2021). Each rat was left in a 20-cm diameter methacrylate cylinder until reaching a total of 20 touches with any of both forelimbs on the walls during exploratory behavior or a maximum of 5-min exploration time. The percentage of contralateral forelimb use with respect to the total number of contacts was calculated.

### 2.5 Electrophysiological recordings

At least 4 weeks after the lesion, electrophysiological procedures were performed as previously described (Antonazzo et al., 2019; Vegas-Suárez et al., 2020; Vegas-Suárez et al., 2021). Animals were anesthetized with urethane (1.2 g/kg, i.p.), and the right jugular vein was cannulated for systemic administration of the drugs. The rat was placed in a stereotaxic frame with its head secured in a horizontal orientation, and body temperature was maintained at ∼37°C for the entire experiment. The skull was exposed, and two 3 mm burr holes were drilled over the right SNr or EP and the ipsilateral motor cortex.

Single-unit extracellular recordings were made by an Omegadot single glass micropipette with a tip diameter of 1–2.5 μm and filled with 2% pontamine sky blue in 0.5% sodium acetate. This electrode was lowered into the motor region of the SNr (AP: −5.3 to −5.8 mm, ML: −2.5 mm, and DV: −7.5 to –8.5 mm for SNr recordings) and EP (relative to bregma and dura, AP: −2.3 to −3 mm, ML: −2.5 mm, and DV: −7 to −8.5 mm). The signal from the recording electrode was amplified with a high-input impedance amplifier (Cibertec SA) and monitored through an oscilloscope and an audio monitor. Neuronal spikes were sampled at 25 kHz and digitized using computer software (CED micro 1401 interface and Spike2 version 7 software, Cambridge Electronic Design, United Kingdom). SNr or EP neurons were identified based on their respective electrophysiological characteristics as previously described (Vegas-Suárez et al., 2020; Vegas-Suárez et al., 2021). To evoke responses in SNr and EP neurons, the motor cortex (relative to bregma and dura, AP: +3.0 to +3.5 mm, ML: −3.2 mm and DV: −1.2 to −1.6 mm) was stimulated at 1 Hz (pulse width, 600 μs; and intensity, 1 mA) using coaxial stainless-steel electrodes (diameter, 250 μm; tip diameter, 100 μm; and tip-to-barrel distance, 300 μm, Cibertec SA) during the recordings. This stimulation protocol does not cause any tissue damage (Antonazzo et al., 2019).

Firing parameters were obtained from the interspike interval histograms, and the firing rate and coefficient of variation (CV; the ratio, expressed as a percentage, between the standard deviation and the mean of the neuron interspike interval) were analyzed offline using the Spike2 software (version 7). The percentage of neurons exhibiting burst firing patterns and burst-related parameters (number of bursts, mean duration of burst, spikes per burst, recurrence of burst, and intraburst frequency) were calculated using a Spike2 script (“surprise.s2s”) based on the Poisson surprise algorithm. Cortically evoked response parameters were analyzed from peristimulus time histograms generated from 180 stimulation trials using 1 ms bins, as previously described (Antonazzo et al., 2019; Antonazzo et al., 2021). The criterion used to determine the existence of an excitatory response was a twofold increase in the standard deviation over the peristimulus period, plus the mean number of spikes, for at least three consecutive bins. The amplitude of excitatory responses was calculated by the difference between the mean number of spikes evoked within the time window of the excitation and the mean number of spikes occurring spontaneously before the stimulation. The duration of an inhibitory response corresponded to the time interval without at least three consecutive bins. We set latency ranges for three cortically evoked responses (i.e., early excitation (EE), inhibition (I), and late excitation (LE)). When the latency was out of the set range or there was no cortically evoked response, neurons were excluded from the analysis of cortically evoked parameters.

### 2.6 Immunohistochemistry and histochemistry assays

Four weeks after the lesion, animals were deeply anesthetized (1.2 g/kg urethane i.p.) and transcardially perfused with saline at 37°C followed by 4% ice-cold paraformaldehyde (PFA) and 0.2% picric acid prepared in a 0.1 M phosphate saline buffer. The brains were removed and fixed overnight in the same PFA solution. Twenty-four hours later, the brains were transferred to a 25% sucrose solution until they sank, and they were serially cut into coronal 40 μm sections using a freezing microtome. Brain slices were kept in a cryoprotectant (glycerol and ethylene glycol in distilled water) solution at −20°C until further processing.

Tyrosine hydroxylase (TH) immunohistochemistry was assessed following our established protocol (Vegas-Suárez et al., 2020; Vegas-Suárez et al., 2021) in the striatum and *substantia nigra*. After endogenous peroxidase inactivation with 3% H_2_0_2_ and 10% methanol in potassium phosphate-buffered saline (KPBS), the sections were preincubated with 5% normal goat serum (NGS) and incubated with primary antibody (rabbit anti-TH, 1:1000, Merck Millipore, Spain) in KPBS/T containing 5% NGS overnight at 22°C. In the following day, the sections were rinsed and incubated for 2 h with the secondary antibody (biotinylated goat anti-rabbit IgG, 1:200, Vector Laboratories, California, United States) in 2.5% NGS KPBS/T. All sections were incubated with an avidin–biotin–peroxidase complex (ABC kit, PK-6100, Vector Laboratories, California, United States) as chromogen, and peroxidase activity was visualized with 0.05% 3,3′-diaminobenzidine (DAB) and 0.03% H_2_O_2_ for 1–2 min. Then, the reaction was stopped by rinsing the sections in KPBS for 5 min.

Cytochrome c oxidase (COX) histochemistry was performed as previously described (Armentero et al., 2006; Blandini et al., 2007; Kaya et al., 2008). Three coronal slices containing the area of interest within the BG and DRN were selected for each subject and placed in incubation medium with 0.05 M PBS, pH 7.4, 1% sucrose, 0.05% nickel sulfate (II), 2.5 µM imidazole (Fluka), 0.025% DAB, and 0.015% cytochrome c (Sigma-Merck). Then, 0.01% catalase (Sigma-Merck) was added to start the enzymatic reaction. COX staining was performed in darkness for 5 h at 37°C. Sections were washed twice with PBS 0.05 M and mounted on gelatine-coated slices. Finally, sections from TH immunohistochemistry or COX histochemistry assays were dehydrated in an ascending series of alcohols, cleared in xylene, and cover slipped with DPX mounting medium.

Quantification of TH expression was assessed as previously described by Ares-Santos et al. (2014). Six serial striatal sections were taken with a ×4 objective of an optic microscope equipped with a Leica DFC290 HD video camera using Analytical Imaging Station software (Imaging Research, Linton, United Kingdom) and NIH-produced software, ImageJ win64 Fiji (https://imagej.net/Fiji), for quantification. First, the threshold was established in the control group, and it was applied to all animals. Then, the area of staining in the striatum was quantified as a proportion of pixels that show staining (stained area) in relation to total pixels (scanned area). Values were expressed as the percentage of stained area of the ipsilateral side with respect to the contralateral one, which was considered as 100%. The SNc was quantified using an optical fractionator provided by Stereo Investigator Program (MicroBrightField Bioscience, Colchester, VT, United States). The region of interest was outlined at ×2.5 magnification and the counting of TH+ immunoreactive neurons was carried out using a ×63 objective. Probes of 50 × 50 μm separated by 100 μm were launched into the previously delimited region, and TH+ neurons inside the probe or crossing on the right side of the *X*–*Y* axis were counted. Values were expressed as the percentage of the TH+ neurons present on the ipsilateral side with respect to the contralateral side. The integrated optical densitometry (IOD) of COX activity in the BG and DRN were measured as grey levels using ImageJ. Digital images were obtained using the ×20 objective of an automatic panoramic digital slide scanner (Pannoramic MIDI II, 3DHistech, Hungary) and the CaseViewer 2.3 (64-bit version) software. The analysis was blinded. Two to four slices were used per nucleus, and the mean IOD was determined by subtracting the background defined as a non-immunoreactive zone.

### 2.7 Data and statistical analysis

Data were analyzed using the computer program GraphPad Prism (v. 5.01, GraphPad Software, Inc.). When more than one neuron was recorded per animal (1–20 neurons), the parameters were averaged per animal. Only one neuron was pharmacologically tested per animal. The average firing rate, CV, and parameters related to cortically evoked responses, and the average TH quantification and COX optical density for each rat were compared between groups using the two-tailed unpaired Student’s t test. Fisher’s exact test was used to assess differences in the number of neurons presenting bursting patterns and cortically evoked response patterns. To evaluate the systemic effect of buspirone (0.6125 and 1.25 mg/kg) or WAY-100635 (0.5 and 1 mg/kg) administration on the firing rate and CV, each dose was administered in dose-dependent manner and analyzed during 90 s after the electrical stimulation. The effects of systemic buspirone were assessed using repeated-measures two-way ANOVA. When allowable, Bonferroni post hoc method was used for correction of multiple comparisons. To evaluate the effect of the drugs on the cortically evoked response, each dose was analyzed through peristimulus time histogram, and a repeated-measures two-way ANOVA followed by Bonferroni’s post hoc test when possible. To analyze the effect on the number of neurons with burst firing, Fisher’s exact test was used. The level of statistical significance was set at *p* < 0.05 with corresponding 95% confidence intervals. Statistical details are shown in Supplementary Table S1.

## 3 Results

### 3.1 Spontaneous discharge and cortically evoked responses of the substantia nigra pars reticulata and entopeduncular neurons

The spontaneous firing rate and cortically evoked responses were recorded in SNr or EP neurons from sham (*n* = 21 and *n* = 21) and 6-OHDA-lesioned animals (*n* = 20 and *n* = 18), respectively. 6-OHDA-lesioned rats showed significant forelimb use asymmetry in the cylinder test (the % of ipsilateral contact with respect to the total number was >70%; *p* < 0.05) and a significant reduction (>80%; *p* < 0.05) in striatal TH immunoreactivity and TH^+^ neurons in the *substantia nigra* in the cerebral hemisphere ipsilateral to the lesion (Supplementary Figure S1). All recorded cells exhibited the typical electrophysiological characteristics of GABAergic SNr or EP neurons (Vegas-Suárez et al., 2020; Vegas-Suárez et al., 2021) (Table 1). However, not all of them responded to motor cortex stimulation; only 66.27% of SNr neurons from the sham group responded compared to 84.54% of SNr neurons from the 6-OHDA group (*p* < 0.05). Neurons in the 6-OHDA-lesioned group, regardless of whether they responded to cortical stimulation, showed significantly higher firing frequency, higher CV, and more burst pattern (*p* < 0.05 for all parameters), in agreement with our previous results (Vegas-Suárez et al., 2020). In the EP, there was no difference in the proportion of neurons that responded to motor cortex stimulation between the sham and 6-OHDA-lesioned groups (76.00 vs. 72.79%, respectively), neither in the firing parameters, as previously shown (Vegas-Suárez et al., 2021). In line with these experiments, we analyzed COX activity as a measurement of the metabolic status of the neurons in basal condition. As already described by others, in 6-OHDA-lesioned animals, COX activity was enhanced in the BG, including the SNr and EP (Supplementary Figure S2), and unmodified in other brain areas as the dorsal raphe nucleus (Supplementary Figure S3).

**TABLE 1:** Electrophysiological characteristics of the spontaneous and cortical-evoked responses in neurons from the sensorimotor circuit of substantia nigra pars reticulata and entopeduncular nucleus in sham and 6-OHDA-lesioned rats.

	SNr			EP	
	Sham (*n* = 21)	6-OHDA (*n* = 20)		Sham (*n* = 21)	6-OHDA (*n* = 18)
No. of responded neurons (%)	66.27	84.54$		76.00	72.79
**Firing rate of total neurons (Hz)**	22.05 ± 1.47	27.08 ± 1.74*		21.54 ± 2.32	21.26 ± 1.33
**CV of total neurons (%)**	39.41 ± 3.00	53.4 5 ± 3.22*		56.34 ± 2.47	60.93 ± 4.11
**Neurons exhibiting burst firing pattern (%)**	**40.92**	71.69#		**63.23**	**56.31**
**Firing rate of responded neurons (Hz)**	22.35 ± 1.58	28.69 ± 1.91^*^		19.42 ± 2.1	23.66 ± 2.77
**CV of responded neurons (%)**	38.68 ± 3.55	52.91 ± 4.87^*^		53.50 ± 3.6	61.00 ± 4.81
**Neurons exhibiting burst firing pattern (%)**	40.46	68.76#		66.92	62.16
**Total no. of neurons showing EE (%)**	79.84	**80.12**		**66.92**	**74.12**
**Latency of EE (ms)**	5.99 ± 0.54	6.55 ± 0.70		5.56 ± 0.61	5.6 ± 0.67
**Duration of EE (ms)**	4.48 ± 0.38	4.55 ± 0.34		5.46 ± 082	4.74 ± 0.39
**Amplitude of EE**	14.32 ± 1.43	14.75 ± 1.33		18.7 ± 1.66	16.57 ± 1.63
**Total no. of neurons showing I (%)**	47.38	**59.80**		**52.42**	**75.28** ^**#**^
**Latency (ms) of I**	15.19 ± 0.87	14.13 ± 0.91		14.8 ± 0.04	13.64 ± 0.65
**Duration (ms) of I**	7.6 ± 0.8	6.48 ± 0.67		6.37 ± 0.71	6.33 ± 0.49
**Total no. of neurons showing LE (%)**	40.46	**81.29** ^**#**^		**57.67**	**79.56** ^**#**^
**Latency (ms) of LE**	24.64 ± 0.80	22.53 ± 0.76		23.63 ± 0.9	22.00 ± 1.07
**Duration (ms) of LE**	6.02 ± 0.53	5.93 ± 0.41		8.42 ± 1.35	6.41 ± 0.6
**Amplitude of LE**	11.46 ± 1.15	12.89 ± 1.17		13.7 ± 1.25	15.07 ± 1.31

Data from the firing rate, the CV, and cortical-evoked response parameters of the mean per animal are expressed as mean ± SEM of n rats. *p < 0.05 (two-tailed unpaired Student’s t test) and ^#^ *p* < 0.05 vs. sham (Fisher’s exact test for burst firing pattern and for percentage of neurons exhibiting the response to cortical stimulation). EE: early excitation; I: inhibition; LE: late excitation. All of the bold values are the total of responsive neurons from the total neurons of each group expressed in percentage.

**FIGURE 1:**
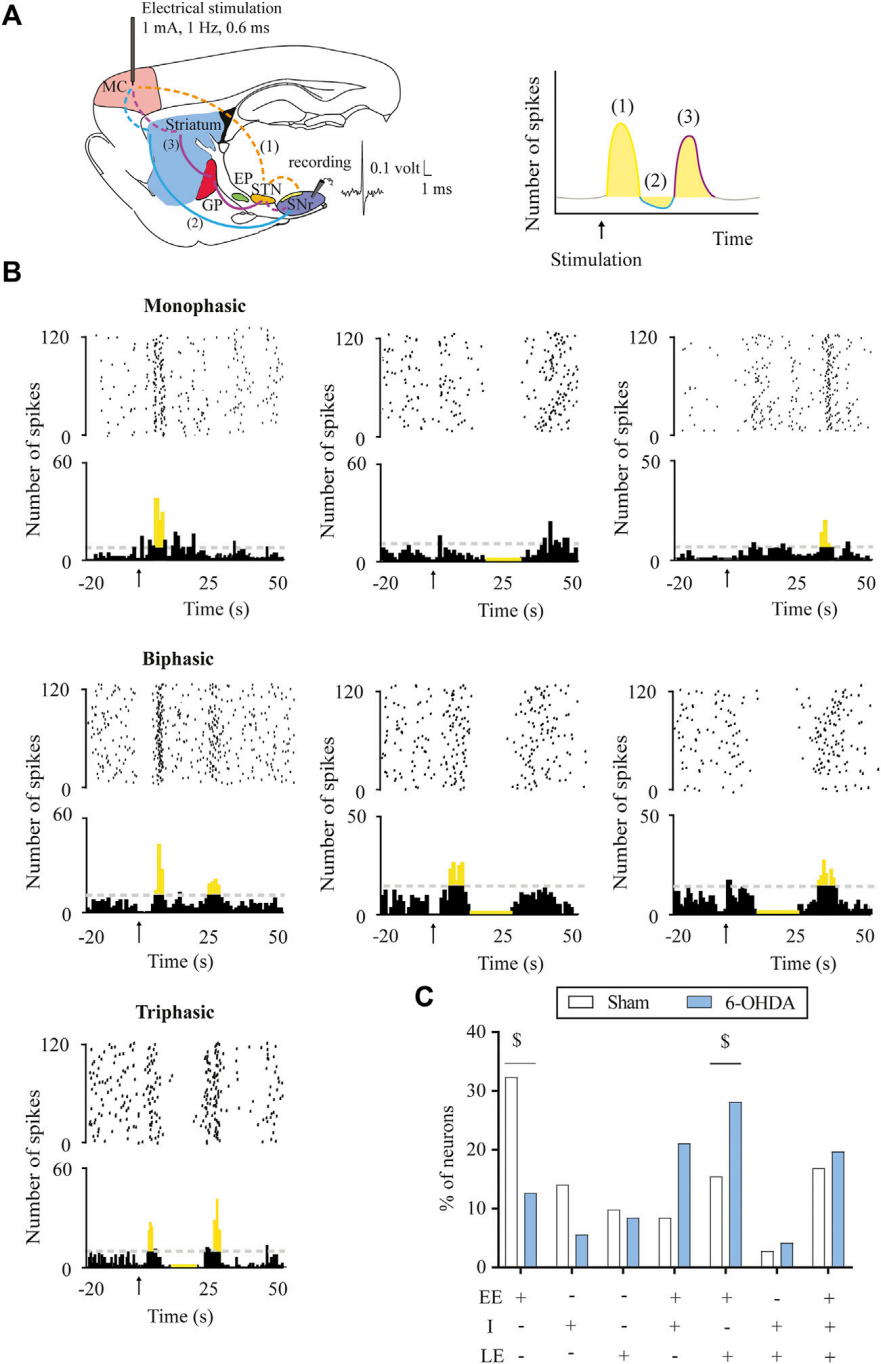
Response patterns evoked in substantia nigra pars reticulata neurons by motor cortex stimulation. **(A)** Left, schematic parasagittal rat brain section illustrating the motor cortex (MC) projections to the BG nuclei (striatum, GPe, STN, and SNr) responsible for each response pattern. Dashed lines represent glutamatergic projections and solid lines, GABAergic projections. On the right, raster plot and peristimulus time histogram showing the characteristic cortically evoked triphasic response in SNr neurons: 1) early excitation (EE) in yellow: activation of hyperdirect pathway (cortex-STN-SNr), 2) inhibition (I) in blue: activation of direct pathway (cortex-striatum-SNr), and 3) late excitation (LE) in red: activation of indirect pathway (cortex-striatum-GPe-STN-SNr). **(B)** Raster plot and peristimulus time histogram showing the different representative examples of a monophasic, biphasic, and triphasic response evoked in a SNr neuron. **(C)** DA loss-induced changes in the percentage of SNr neurons displaying different patterns of response: the proportion of neurons displaying monophasic (i.e., early excitation) responses was reduced, in favour of more biphasic (i.e., early excitation + late excitation) responses in the 6-OHDA-lesioned group. Data are expressed as percentages. ^$^ *p* < 0.05 (Fisher’s exact test).

**FIGURE 2:**
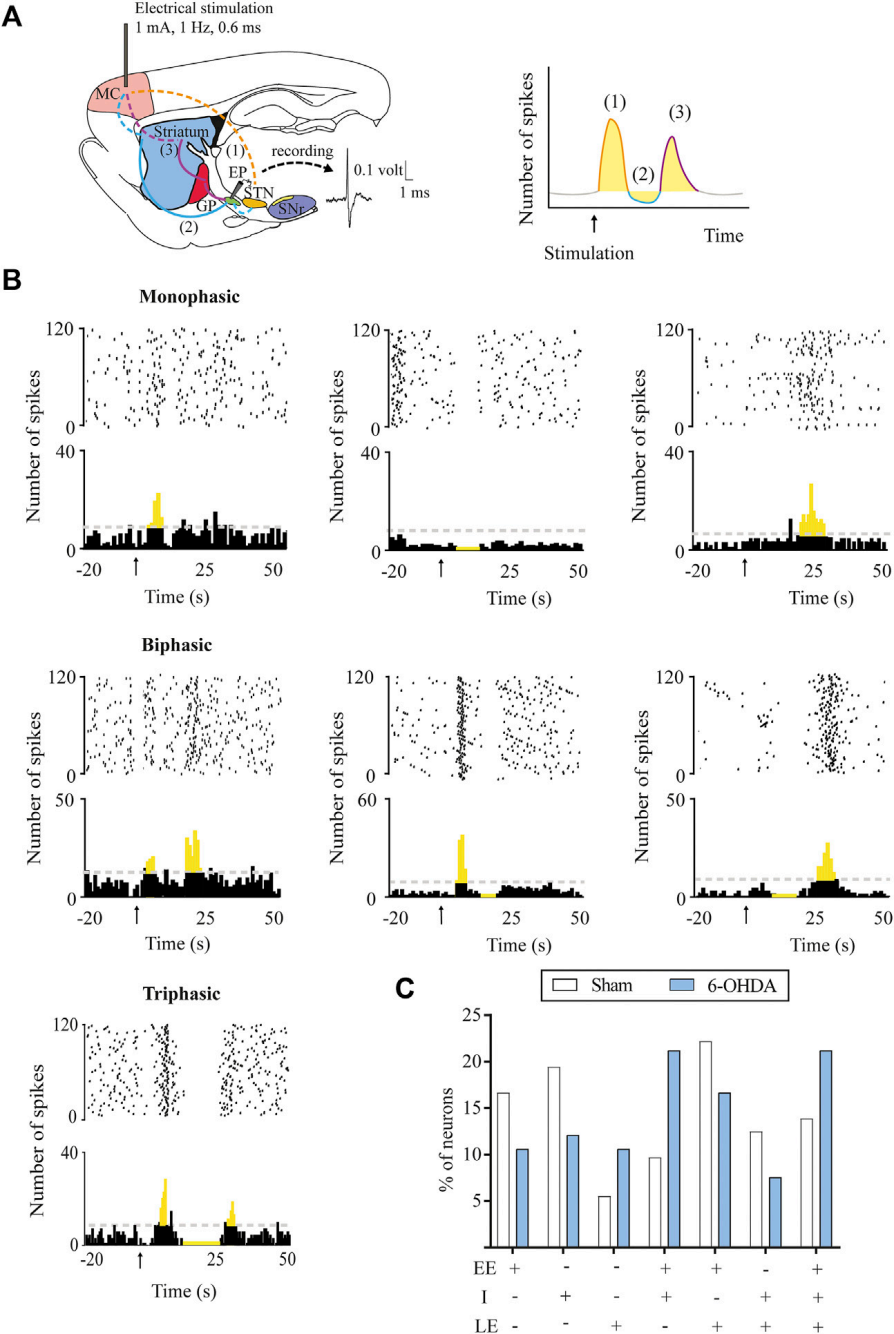
Response patterns evoked in entopeduncular neurons by motor cortex stimulation. **(A)** Left, schematic parasagittal rat brain section illustrating the motor cortex (MC) projections to the BG nuclei (striatum, GPe, STN, and EP) responsible for each response pattern. Dashed lines represent glutamatergic projections and solid lines, GABAergic projections. On the right, raster plot and peristimulus time histogram showing the characteristic cortically evoked triphasic response in EP neurons: 1) early excitation (EE) in yellow: activation of hyperdirect pathway (cortex-STN-SNr), 2) inhibition (I) in blue: activation of direct pathway (cortex-striatum-EP), and 3) late excitation (LE) in red: activation of indirect pathway (cortex-striatum-GPe-STN-EP). **(B)** Raster plot and peristimulus time histogram showing the different representative examples of a monophasic, biphasic, and triphasic response evoked in an EP neuron. **(C)** DA loss did not alter the percentage of SNr neurons displaying different patterns of response monophasic, biphasic, or triphasic in the 6-OHDA-lesioned group. Data are expressed as percentages.

The response evoked by the cortical stimulation in SNr and EP neurons consisted of a combination of an early excitation, inhibition, and/or late excitation. According to Maurice et al. (1999) and Nambu et al. (2000), early excitation involves the activation of the cortico-SNT-SNr/EP pathway, inhibition involves the activation of the cortico-striato-SNr/EP pathway, and late excitation involves the activation of the cortico-striato-GPe-STN-SNr/EP pathway (Figures 1A, 2A). As exemplified in Figures 1B, 2B, three different patterns of response were found according to the activation of the different pathways along the circuits: 1) monophasic response (early excitation, inhibition, or late excitation), 2) biphasic response (combination of two monophasic responses), and 3) triphasic response. The percentage of occurrence of these patterns was significantly altered by the 6-OHDA lesion, leading to a lower percentage of monophasic early excitation and a higher percentage of biphasic early and late excitation patterns in the SNr (*p* < 0.05) (Figure 1C). No changes were observed in EP neurons (Figure 2C). The parameters analyzed for each of the responses, such as latency of appearance, duration, and amplitude of the excitations, were not altered by the 6-OHDA lesion. However, the total number of neurons exhibiting late excitation was higher in the SNr, while the total number of neurons exhibiting inhibition and late excitation was higher in the EP after the lesion with 6-OHDA (Table 1).

### 3.2 Effect of 5-HT_1A_ receptor drugs on the cortically evoked responses of substantia nigra pars reticulata

The effect of the partial 5-HT_1A_ receptor agonist, buspirone, on motor cortico-SNr was studied by comparing the cortically evoked responses before and after drug administration. In SNr neurons from sham (*n* = 14) and 6-OHDA-lesioned (*n* = 13) rats, buspirone (0.6125 and 1.25 mg/kg, i.v.) did not modify the basal firing rate or the CV and had little effect on burst activity (*p* < 0.05), according to our published results (Vegas-Suárez et al., 2020) (Supplementary Table S2). Posterior administration of the 5-HT_1A_ receptor antagonist, WAY-100635 (0.5 mg/kg, i.v.), did not cause any effect on SNr neuron activity in none of the studied groups.

**FIGURE 3:**
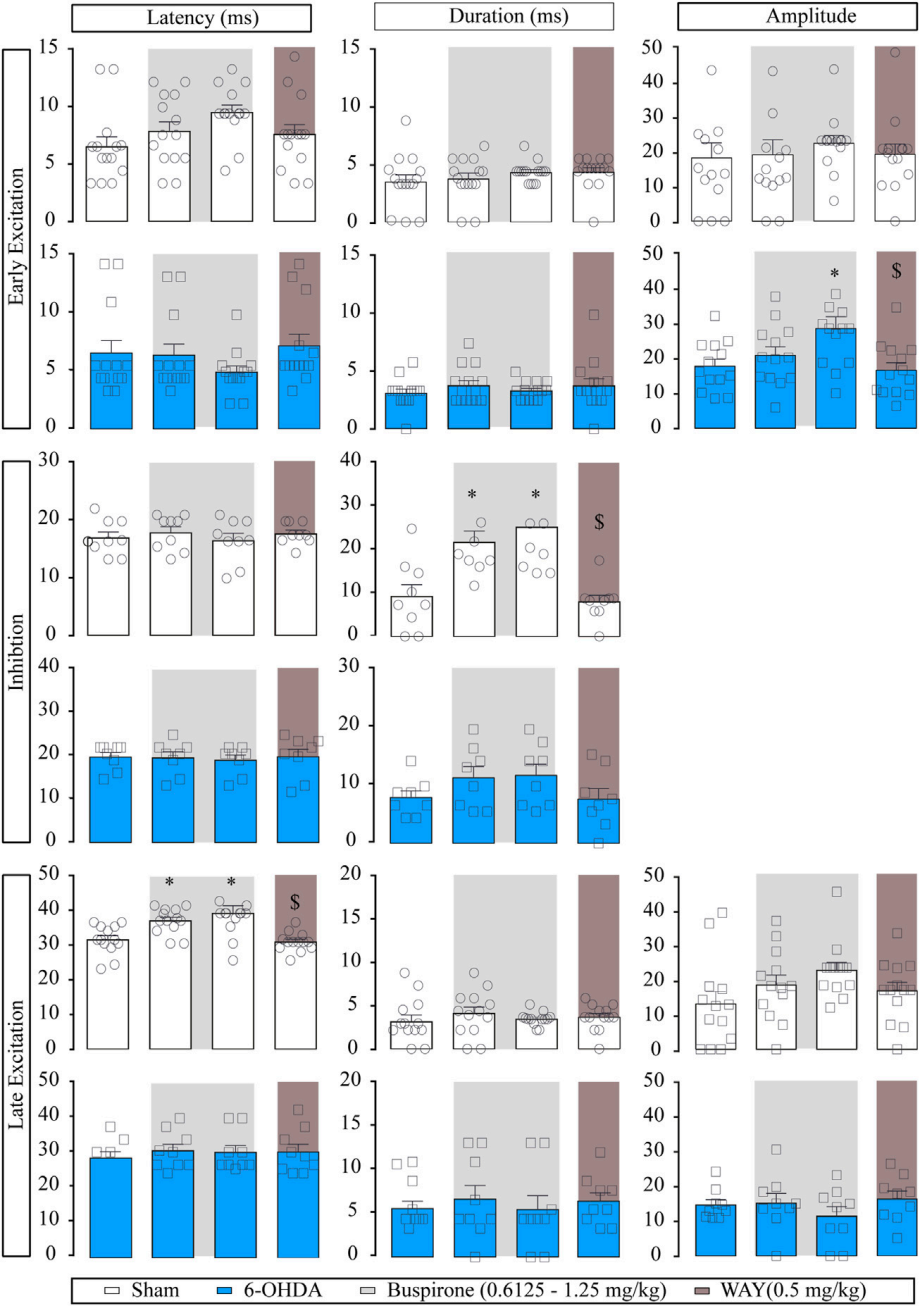
Effect of the systemic administration of buspirone on motor cortico-nigral information. Representation of characteristic parameters related to cortically evoked responses (latency, duration, and amplitude of the responses). The latency, duration, and amplitude of the early excitatory component are on the top (sham *n* = 11, 6-OHDA *n* = 13). The latency and duration of the inhibitory component are in the middle (sham *n* = 7, 6-OHDA *n* = 8). The latency, duration, and amplitude of the late excitatory component are on the bottom (sham *n* = 10, 6-OHDA *n* = 9). Note that buspirone enhanced the duration of inhibition in the sham group and delayed the latency of late excitation. Each bar represents the mean ± S.E.M. Each dot represents the value from one neuron before and after buspirone (0.6125 and 1.25 mg/kg, i.v.) in light grey and WAY-100635 (0.5 mg/kg i.v.) in dark grey. *p < 0.05 vs. respective baseline; and ^$^ *p* < 0.05 vs. buspirone (RM two-way ANOVA followed by Bonferroni’s post hoc test).

Regarding the evoked responses of SNr neurons, as summarized in Figure 3, in the sham group, both doses of buspirone significantly enhanced the duration of the inhibition (*p* < 0.05) and increased the latency of the late excitation (*p* < 0.05). In the 6-OHDA group, buspirone (1.25 mg/kg) significantly increased the amplitude of the early excitation (*p* < 0.05).

**FIGURE 4:**
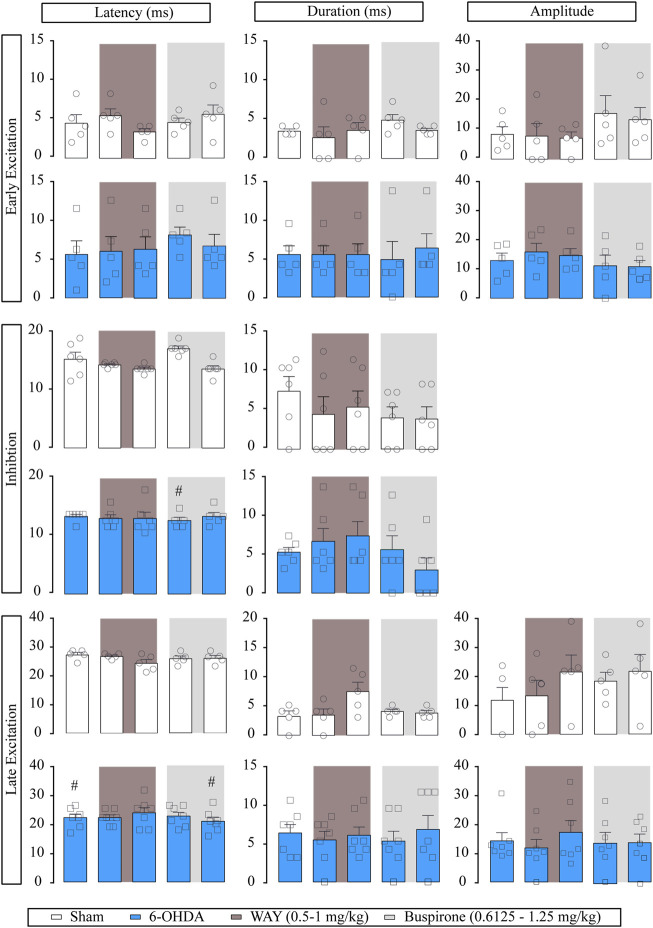
Effect of the systemic administration of WAY-100635 on motor cortico-nigral information. Representation of characteristic parameters related to cortically evoked responses (latency, duration, and amplitude of the responses). The latency, duration and amplitude of the early excitatory component are on the top (sham *n* = 5, 6-OHDA *n* = 6). The latency and duration of the inhibitory component are in the middle (sham *n* = 6, 6-OHDA *n* = 6). The latency, duration, and amplitude of the late excitatory component are on the bottom (sham *n* = 5, 6-OHDA *n* = 7). Each bar represents the mean ± SEM. Each dot represents the value from one neuron before and after WAY-100635 (0.5–1 mg/kg i.v.) and buspirone (0.6125 and 1.25 mg/kg, i. v.) in dark grey and in light grey. ^#^ *p* < 0.05 vs. sham (RM two-way ANOVA followed by Bonferroni’s post hoc test).

For better studying if the effect of buspirone was due to 5-HT_1A_ activation, in another set of experiments, we administered the selective 5-HT_1A_ receptor antagonist WAY-100635 (0.5 and 1 mg/kg, i.v.) prior to buspirone. Neither of the administered doses modified the basal firing rate or the CV and had little effect on burst firing pattern (*p* < 0.05) in SNr from both sham (*n* = 6) and 6-OHDA-lesioned rats (*n* = 7). Posterior buspirone administration (0.6125 and 1.25 mg/kg, i.v., respectively) did not have any effect on SNr neuron activity (Supplementary Table S4). Regarding the effect of WAY-100635 on the evoked responses of SNr neurons, WAY-100635 failed to modify the cortico-SNr-evoked response (Figure 4). Posterior buspirone administration did not show any effect. All statistical details are shown in Supplementary Table S1.

### 3.3 Effect of 5-HT_1A_ receptor drugs on the cortically evoked responses of the entopeduncular neurons

In the EP from the sham animals (*n* = 13), buspirone (0.6125 mg/kg) significantly decreased the neuron firing rate (*p* < 0.05), while the 6-OHDA-lesioned group (*n* = 10) showed smaller number of EP cells exhibiting a burst firing pattern (Supplementary Table S3). In order to avoid altering basal conditions, a higher dose of buspirone was not used on EP neurons.

**FIGURE 5:**
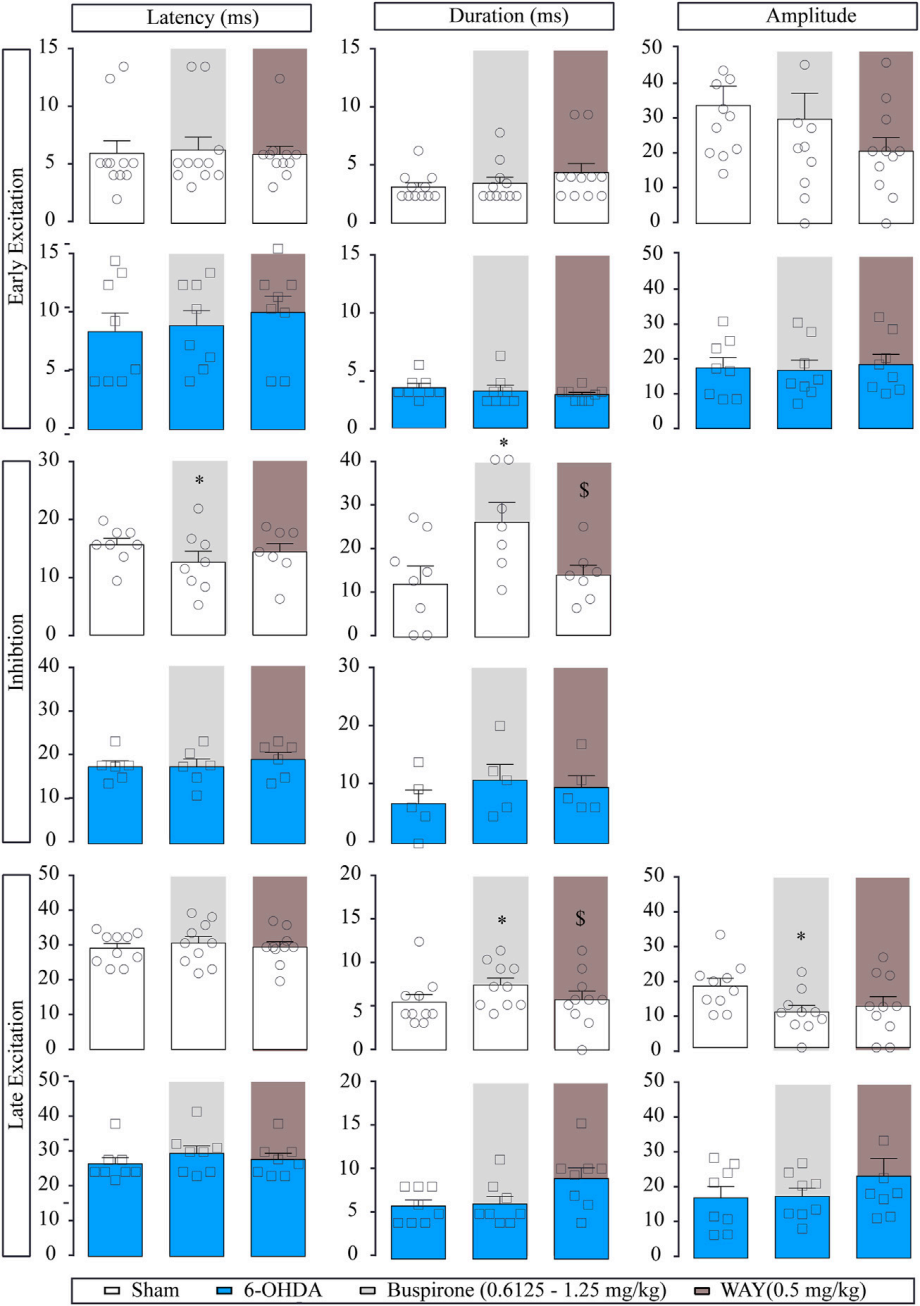
Effect of the systemic administration of buspirone on motor cortico-entopeduncular information. Representation of the characteristic parameters related to cortically evoked responses (latency, duration, and amplitude of the responses). The parameters of the early excitatory component are on the top (sham *n* = 11, 6-OHDA *n* = 8). The parameters of the inhibitory component are in the middle (sham *n* = 7, 6-OHDA *n* = 6). The parameters of the late excitatory component are on the bottom (sham *n* = 10, 6-OHDA *n* = 8). Note that buspirone enhanced the duration of inhibition and late excitation in the sham group. Each bar represents the mean ± SEM of n rats. Each dot represents the value from one neuron before and after buspirone (0.6125 mg/kg, i.v.) and WAY-100635 (0.5 mg/kg). *p < 0.05 vs. respective baseline and ^$^ *p* < 0.05 vs. buspirone (RM two-way ANOVA followed by Bonferroni’s post hoc test).

Similar to what we have described for SNr neurons, the effect of buspirone on cortico-EP transmission was modified by DA loss (Figure 5). Thus, in the sham group, buspirone increased the duration (*p* < 0.05) and reduced the latency (*p* < 0.05) of the inhibitory component of the cortically evoked response of EP neurons. In addition, the duration of the late excitation was significantly higher (*p* < 0.05), and its amplitude was reduced (*p* < 0.05) after buspirone administration. These effects were reversed by WAY-100635. In contrast to that observed in the sham group, in 6-OHDA-lesioned animals, buspirone had no effect on cortico-EP transmission.

**FIGURE 6:**
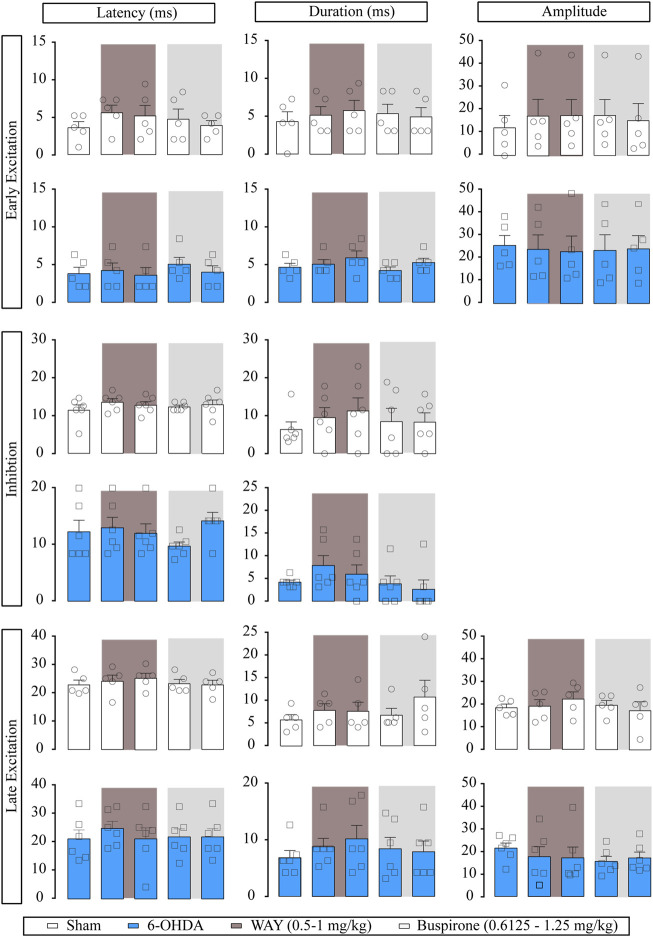
Effect of the systemic administration of WAY-100635 on motor cortico-entopeduncular information. Representation of characteristic parameters related to cortically evoked responses (latency, duration, and amplitude of the responses). The latency, duration, and amplitude of the early excitatory component are on the top (sham *n* = 5, 6-OHDA *n* = 5). The latency and duration of the inhibitory component are in the middle (sham *n* = 6, 6-OHDA *n* = 6). The latency, duration, and amplitude of the late excitatory component are on the bottom (sham *n* = 5, 6-OHDA *n* = 6). Each bar represents the mean ± SEM. Each dot represents the value from one neuron before and after WAY-100635 (0.5–1 mg/kg i.v.) and buspirone (0.6125 and 1.25 mg/kg, i.v.) in dark grey and in light grey, respectively.

In a separate set of experiments, we administered the 5-HT_1A_ receptor antagonist prior to buspirone. WAY-100635 (0.5 and 1 mg/kg, i.v.) administered alone did not modify the basal firing rate or the CV and had little effect on burst firing pattern (*p* < 0.05) in EP neurons from both sham (*n* = 6) and 6-OHDA-lesioned rats (*n* = 7). Posterior buspirone administration (0.6125 and 1.25 mg/kg, i.v., respectively) did not have any effect on EP neuron activity (Supplementary Table S5). In the cortico-EP transmission, WAY-100635 had no effect either in sham or 6-OHDA groups (Figure 6). Posterior buspirone administration did not show any effect. All statistical details are shown in Supplementary Table S1.

## 4 Discussion

In this study, we analyzed the effect of buspirone on BG output nuclei, focusing on cortico-SNr and cortico-EP transmission in sham and 6-OHDA-lesioned rats. The output nuclei of the BG are interesting as genetic and pharmacological manipulation of these nuclei contribute to improve motor disability in PD (Olanow et al., 2000; Weiss et al., 2013; Assaf and Schiller, 2019), probably due to their projections to the thalamic nuclei (Lanciego et al., 2012). It is well known that lack of DA triggers changes in the BG output nuclei affecting not only neuron basal activity (Tseng et al., 2000; Ruskin et al., 2002; Meissner et al., 2006; Lacombe et al., 2009; Aristieta et al., 2016, 2019; Vegas-Suárez et al., 2020; Vegas-Suárez et al., 2021), but also their response to cortical stimulation (Belluscio et al., 2007; Kita and Kita, 2011; Janssen et al., 2017; Antonazzo et al., 2019; Antonazzo et al., 2021; Sano and Nambu, 2019; Wahyu et al., 2021). Here, we also provide evidence of how 5-HT_1A_ compounds modulate this transmission. In our study, buspirone differentially modulates cortical response in the SNr/EP in control and 6-OHDA-lesioned animals.

This work was developed in a well-characterized model of DA deficit that used the toxin 6-OHDA to induce unilateral DA loss. In this experimental model, we have previously shown that the activity of SNr and EP neurons displays high level of burst activity and more irregular firing pattern (Aristieta et al., 2016, 2019; Vegas-Suárez et al., 2020; Vegas-Suárez et al., 2021). Similar findings were observed in the present study, but in addition, EP neurons that responded to cortical stimulation showed higher firing rate after DA loss. This may be due to the heterogeneity of the EP nucleus, which contains at least three classes of neurons (Wallace et al., 2017) and receives afferents from several cortical areas (Naito and Kita, 1994). As also seen by other investigators, 6-OHDA-lesioned animals showed increased COX activity in all BG structures but not in the DRN, in concordance with the electrophysiological changes produced by the DA depletion (Kita and Kita, 2011; Miguelez et al., 2011; Aristieta et al., 2012; Aristieta et al., 2016; Aristieta et al., 2019; Suarez et al., 2016; Suarez et al., 2018; Suarez et al., 2020).

Electrical stimulation of the motor cortex induced a similar response pattern in the SNr and EP, including the monophasic, biphasic, and triphasic responses, as described in awake or anesthetized rodents (Degos et al., 2005; Aliane et al., 2009; Beyeler et al., 2010; Lagière et al., 2013; Chiken et al., 2015; Antonazzo et al., 2019; Antonazzo et al., 2021). Furthermore, the response parameters were compatible with previous data obtained from anesthetized rats for SNr (Beyeler et al., 2010; Antonazzo et al., 2019; Antonazzo et al., 2021) and EP neurons (Kita and Kita, 2011). The latency, duration, and amplitude of the response were similar for SNr and EP neurons, suggesting that the cortical stimulus flows to both nuclei in a similar way. Several similarities were also found in the changes induced by DA loss in the output BG neuronal response to cortical stimulation.

It is well known that DA is a crucial modulator of the striatal processing of cortical signals (Surmeier et al., 2007), and consequently, information transmission of cortical-BG output nuclei is altered by DA loss. Consistent with this, our results show that in the SNr, 6-OHDA lesion caused a decrease in the percentage of neurons showing early excitatory monophasic response, which is associated with the hyperdirect pathway activation. In the same conditions, we found increased percentage of neurons with biphasic response, consisting of early and late excitation, which involves the hyperdirect and the indirect pathways. The results obtained in 6-OHDA-lesioned rats also showed changes in the parameters that define the cortical stimulation response compatible with an increase of the components involving the STN, but there were some differences between the patterns of changes. These differences may be due to methodological aspects, type of anesthesia used, and intensity of stimulation of electrode placement coordinates. Antonazzo et al. (2021) reported a decrease in triphasic response and biphasic (inhibition plus late excitation) response and an increase in the early excitation in the SNr neurons of chloral hydrate-anesthetized rats, and Wahyu et al. (2021) described that monophasic early or late excitation and biphasic excitation became dominant in 6-OHDA-lesioned awake mice. Kita and Kita (2011) found that while cortical stimulation induced a triphasic response in 6-OHDA-lesioned rats, most neurons showed early excitation and long inhibition; however, in this case, they used isoflurane to induce a lower degree of anesthesia than that used in the present study. Additionally, recordings in the SNr from awake-6-OHDA-lesioned mice indicated that monophasic excitation became dominant, and the evoked inhibition was suppressed (Wahyu et al., 2021). Other studies further stress that modification in the pattern of response in the SNr exclusively affects those neurons with pathological low frequency oscillations (Belluscio et al., 2007). Despite some differences between the mentioned studies, it seems that the response of the components involving the STN are consistently more affected by DA loss, which leads to a prevalence of the hyperdirect and indirect pathways.

More interestingly, we analyzed the effect of buspirone on the cortical stimulation-evoked response of the BG output nuclei since this drug has shown not only antidyskinetic and anxiolytic effects in parkinsonian conditions, but also variable influence on motor performance. In parkinsonian animals with dyskinesia or patients with PD, buspirone administration worsens motor function (Dekundy et al., 2007; Lindenbach et al., 2015; Rissardo and Caprara, 2020). However, in other models, as those treated with haloperidol, buspirone improves motor coordination and catalepsy (Nayebi and Sheidaei, 2010). In control conditions, at the doses used in this study, buspirone shows anxiolytic effect without worsening or even enhancing motor function (Siemiatkowski et al., 2000; Pogorelov et al., 2007). In line with these results, our previous electrophysiological studies have shown that the effect of buspirone changes in DA-depleted animals, not only in SNr and in EP neurons (it reduces the firing rate in control conditions and decreases burst activity after DA loss) (Vegas-Suárez et al., 2020; Vegas-Suárez et al., 2021) but also in STN neurons (Sagarduy et al., 2016). Similarly, the analysis of the evoked response parameters of SNr neurons to cortical stimulation revealed that the effect of systemic administration of buspirone was altered by DA loss. In control animals we observed that buspirone potentiates transmission in the direct pathway as longer duration of inhibition was seen. This activation may increase GABA release in the output BG nuclei with a subsequent disinhibition of the thalamocortical circuits and promotion of motor activity. In 6-OHDA-lesioned animals, buspirone increased the amplitude of the early excitation indicating potentiation of the hyperdirect pathway, which can be related to motor impairment (Gerfen and Surmeier, 2011). In this sense, a recent publication has demonstrated that elimination of the hyperdirect cortico-subthalamic pathways induces motor hyperactivity in mice (Koketsu et al., 2021). Similarly, to SNr, in EP neurons, the parameter related to the direct pathway was modified in the sham group. When WAY-100635 was systemically administered prior to buspirone in both nuclei, this 5-HT_1A_ receptor antagonist did not alter electrophysiological parameters and blocked the observed effect of buspirone on cortical-evoked responses, confirming the involvement of this receptor.

Buspirone modulates the direct trans-striatal via activation of presynaptic 5-HT_1A_ receptors in the DRN. This subtype receptor is expressed in most basal ganglia nuclei, and slight or no modifications have been described in 6-OHDA-lesioned animals (Miguelez et al., 2014). In control conditions, 5-HT exerts an inhibitory influence on dopaminergic nigrostriatal neurotransmission by inhibiting adenylate cyclase activity that activates potassium and inhibits calcium channels (Harrington et al., 1988). Therefore, activation of somatodendritic 5-HT_1A_ receptors increases DA release in different areas of the BG (Haleem, 2015) and facilities movement. On the contrary, buspirone can also activate postsynaptic 5-HT_1A_ receptors in the BG nuclei and thalamus, promoting motor activity (Mignon and Wolf, 2007; Nayebi and Sheidaei, 2010; Shimizu et al., 2013). *In vivo* microdialysis studies have revealed that 5-HT_1A,_ agonists including buspirone can evoke different effects depending on the brain region (Matos et al., 1996; Gobert et al., 1999; Silverstone et al., 2012) and the level of DA integrity (Azkona et al., 2014; Sagarduy et al., 2016; Vegas-Suárez et al., 2020, Vegas-Suárez et al., 2021). In control animals or those with a high degree of nigral preservation, such as haloperidol-treated rats, buspirone facilitates movement. In 6-OHDA-lesioned animals with severe dopaminergic loss, buspirone fails to increase DA release in the striatum and to modify glutamate and GABA levels in the SNr (Vegas-Suárez et al., 2020). In this condition, the major effect will be that due to the activation of postsynaptic receptors. It would be interesting to investigate how those postsynaptic receptors changes in parkinsonian conditions, as changes in those receptors may help to understand our results.

One limitation of the present work is that electrophysiological recordings were performed in urethane-anesthetized rats, and it may be argued that this does not accurately represent clinical situations. It is widely recognized that urethane induces a long-lasting steady level of anesthesia with minimal effects on autonomic and cardiovascular systems (Hara and Harris, 2002; Neville and Haberly, 2003), and this anesthetic induces prolonged maintenance of a stable brain state (Steriade, 2006). Interestingly, the burst activity after 6-OHDA lesion is still found more enhanced under anesthesia than in the awake state (Magill et al., 2001; Tseng et al., 2001; Lobb and Jaeger, 2015). From the experimental point of view, it would be interesting to study the effect of buspirone in freely moving or awake animals because it is closer to physiological conditions; however, this approach is tedious and does not allow to evaluate the intravenous immediate effect of the drugs. Using multi-electrode recording technology could increase the robustness of the obtained DA data.

Altogether, we show or we report the effect of buspirone in cortical information transmission from parkinsonian rats can contribute to understand the electrophysiological changes of the BG output nuclei in DA-depleted conditions and the response to potential therapeutic drugs. The present findings may be useful in the development of new treatments for movement disorders such as PD that affect a considerable number of people and are devoid of effective treatments.

## Data Availability

The original contributions presented in the study are included in the article/Supplementary Material; further inquiries can be directed to the corresponding authors.

## Abbreviations

5-HT, serotonin, 5-hydroxytryptamine; 6-OHDA, 6-hydroxydopamine; BG, Basal ganglia; CV, coefficient of variation; DA, dopamine, dopaminergic; DRN, dorsal raphe nucleus, dorsal region; EP, entopeduncular nucleus; GPe, external *globus pallidus*; GPi, internal *globus pallidus*; PD, Parkinson’s disease; STN, subthalamic nucleus; SNr, *substantia nigra pars reticulata*.
