# Supplementary material for: Motor cortico-nigral and cortico-entopeduncular information transmission and its modulation by buspirone in control and after dopaminergic denervation
Source: Front Pharmacol. 2022 Aug 30;13:953652. doi: 10.3389/fphar.2022.953652 (PMC9483552; doi:10.3389/fphar.2022.953652)
Supplement: Supplementary file 1 [file DataSheet1.docx]

**SUPPLEMENTARY MATERIAL**

**Dopamine loss disturbs motor cortico-nigral and motor cortico-entopeduncular transmission and its modulation by buspirone**

Sergio Vegas-Suárez^1,2,3,4,*,^ Teresa Morera-Herreras^1,2^, Catalina Requejo^3,4^, José Vicente Lafuente^5^, Rosario Moratalla^3,4^, Cristina Miguélez^1,2,*,LA^ and Luisa Ugedo^1,2,LA^

1 Department of Pharmacology, Faculty of Medicine and Nursing, University of the Basque Country (UPV/EHU), 48940 Leioa, Spain

2 Autonomic and Movement Disorders Unit, Neurodegenerative Diseases, Biocruces Health Research Institute, Barakaldo, Bizkaia, Spain

3 Cajal Institute, Spanish National Research Council (CSIC), Madrid, Spain.

4 Network Center for Biomedical Research in Neurodegenerative Diseases (CIBERNED), Carlos III Institute of Health (ISCIII), Madrid, Spain

5 LaNCE, Department of Neuroscience, University of the Basque Country (UPV/EHU), Leioa, Spain

LA C Miguelez and L Ugedo should be considered shared last authors

* Corresponding authors:

**CONTENTS:**

**Supplementary Tables (5) and Figures (3).**

*^*^* Corresponding authors:

**Cristina Miguélez, Ph.D.**

Department of Pharmacology, Faculty of Medicine and Nursing, University of the Basque Country (UPV/EHU), 48940 Leioa, SPAIN

E-mail address: cristina.miguelez@ehu.eus

Postal address: Department of Pharmacology, Faculty of Medicine and Nursing, University of the Basque Country (UPV/EHU), Barrio Sarriena s/n, 48940-Leioa, Spain

Phone number: +34 94 601 601 56 37

ORCID: 0000-0001-6624-2932

**Sergio Vegas-Suárez, Ph.D.**

Department of Pharmacology, Faculty of Medicine and Nursing, University of the Basque Country (UPV/EHU), 48940 Leioa, SPAIN

E-mail address: sergio.vegas@ehu.eus

Postal address: Department of Pharmacology, Faculty of Medicine and Nursing, University of the Basque Country (UPV/EHU), Barrio Sarriena s/n, 48940-Leioa, Spain

Phone number: +34 94 601 33 15

ORCID: 0000-0001-5925-189X

**Luisa Ugedo, MD, Ph.D.**

Department of Pharmacology, Faculty of Medicine and Nursing, University of the Basque Country (UPV/EHU), 48940 Leioa, SPAIN

E-mail address: luisa.ugedo@ehu.eus

Postal address: Department of Pharmacology, Faculty of Medicine and Nursing, University of the Basque Country (UPV/EHU), Barrio Sarriena s/n, 48940-Leioa, Spain

Phone number: +34 94 601 55 74

ORCID: 0000-0001-5993-0606

**Supplementary Table 1. Statistical Details**

| **Effect of buspirone on electrophysiological parameters in SNr neurons** | | | | |
| --- | --- | --- | --- | --- |
| **RM two-way ANOVA** | **Treatment**  **F_(DFn, DFd)_** | **p value** | **Lesion**  **F_(DFn, DFd)_** | **p value** |
| **Firing Rate** | F _(2, 52)_ = 0.2636 | p > 0.05 | F _(1, 26)_ = 0.09252 | p > 0.05 |
| **CV** | F _(2, 52)_ = 0.6556 | p > 0.05 | **F _(1, 26)_ = 10.31** | **p < 0.05** |
| **Reversion by WAY-100635** | | | | |
| **RM two-way ANOVA** | **Treatment**  **F_(DFn, DFd)_** | **p value** | **Lesion**  **F_(DFn, DFd)_** | **p value** |
| **Firing Rate** | **F_(1, 26)_ = 6.514** | **p < 0.05** | F_(1, 26)_ = 0.1812 | p > 0.05 |
| **CV** | F_(1, 26)_ = 0.00297 | p > 0.05 | **F_(1, 26)_ = 10.63** | **p < 0.05** |
| **Effect of buspirone on cortically evoked activity parameters in SNr neurons** | | | | |
| **RM two-way ANOVA** | **Treatment**  **F_(DFn, DFd)_** | **p value** | **Lesion**  **F_(DFn, DFd)_** | **p value** |
| **Latency of EE** | F_(2, 50)_ = 1.03 | p > 0.05 | F_(1, 25)_ = 3.665 | p > 0.05 |
| **Duration of EE** | F_(2, 50)_ = 1.701 | p > 0.05 | F_(1, 25)_ = 1.741 | p > 0.05 |
| **Amplitude of EE** | **F_(2, 50)_ = 3.761** | **p < 0.05** | F_(1, 25)_ = 0.7174 | p > 0.05 |
| **Latency of I** | F_(2, 30)_ = 2.023 | p > 0.05 | F_(1, 15)_ = 3.32 | p > 0.05 |
| **Duration of I** | **F _(2, 30)_ = 16.74** | **p < 0.05** | F_(1, 15)_ = 2.082 | p > 0.05 |
| **Latency of LE** | **F _(2, 40)_ = 7.40** | **p < 0.05** | **F_(1, 20)_ = 9.169** | **p < 0.05** |
| **Duration of LE** | F_(2, 40)_ = 1.154 | p > 0.05 | F_(1, 20)_ = 0.2308 | p > 0.05 |
| **Amplitude of LE** | F_(2, 40)_ = 1.154 | p > 0.05 | F_(1, 20)_ = 0.2308 | p > 0.05 |
| **Reversion by WAY-100635** | | | | |
| **RM two-way ANOVA** | **Treatment**  **F_(DFn, DFd)_** | **p value** | **Lesion**  **F_(DFn, DFd)_** | **p value** |
| **Latency of EE** | **F_(1, 25)_ = 5.088** | **p < 0.05** | F_(1, 25)_ = 0.0075 | p > 0.05 |
| **Duration of EE** | **F_(1, 25)_ = 4.302** | **p < 0.05** | F_(1, 25)_ = 2.335 | p > 0.05 |
| **Amplitude of EE** | F_(1, 25)_ = 0.00145 | p > 0.05 | F_(1, 25)_ = 0.1326 | p > 0.05 |
| **Latency of I** | F_(1, 15)_ = 0.2607 | p > 0.05 | F_(1, 15)_ = 0.4759 | p > 0.05 |
| **Duration of I** | F_(1, 15)_ = 0.9005 | p > 0.05 | F_(1, 15)_ = 3.55 | p > 0.05 |
| **Latency of LE** | F_(1, 20)_ = 0.2596 | p > 0.05 | F_(1, 20)_ = 0.6376 | p > 0.05 |
| **Duration of LE** | F_(1, 20)_ = 1.159 | p > 0.05 | F_(1, 20)_ = 0.7138 | p > 0.05 |
| **Amplitude of LE** | F_(1, 20)_ = 1.916 | p > 0.05 | F_(1, 20)_ = 1.66 | p > 0.05 |
| **Effect of buspirone on electrophysiological parameters in EP neurons** | | | | |
| **RM two-way ANOVA** | **Treatment**  **F_(DFn, DFd)_** | **p value** | **Lesion**  **F_(DFn, DFd)_** | **p value** |
| **Firing Rate** | **F _(1, 20)_ = 8.035** | **p < 0.05** | **F _(1, 20)_ = 9.67** | **p < 0.05** |
| **CV** | F _(1, 20)_ = 0.129 | p > 0.05 | F _(1, 20)_ = 1.717 | p > 0.05 |
| **Reversion by WAY-100635** | | | | |
| **RM two-way ANOVA** | **Treatment**  **F_(DFn, DFd)_** | **p value** | **Lesion**  **F_(DFn, DFd)_** | **p value** |
| **Firing Rate** | F_(1, 20)_ = 0.9075 | p > 0.05 | F_(1, 20)_ = 1.461 | p > 0.05 |
| **CV** | F_(1, 20)_ = 0.0344 | p > 0.05 | F_(1, 20)_ = 0.009 | p > 0.05 |
| **Effect of buspirone on cortically evoked activity parameters in EP neurons** | | | | |
| **RM two-way ANOVA** | **Treatment**  **F_(DFn, DFd)_** | **p value** | **Lesion**  **F_(DFn, DFd)_** | **p value** |
| **Latency of EE** | F_(1, 17)_ = 3.901 | p > 0.05 | F_(1, 17)_ = 2.011 | p > 0.05 |
| **Duration of EE** | F_(1, 17)_ = 0.0002 | p > 0.05 | F_(1, 17)_ = 0.0258 | p > 0.05 |
| **Amplitude of EE** | F_(1, 17)_ = 0.5007 | p > 0.05 | F_(1, 17)_ = 3.947 | p > 0.05 |
| **Latency of I** | **F _(1, 12)_ = 5.991** | **p < 0.05** | F _(1, 12)_ = 0.034 | p > 0.05 |
| **Duration of I** | **F _(1, 12)_ = 37.92** | **p < 0.05** | F _(1, 12)_ = 1.943 | p > 0.05 |
| **Latency of LE** | F_(1, 16)_ = 3.551 | p > 0.05 | F_(1, 16)_ = 0.5897 | p > 0.05 |
| **Duration of LE** | **F _(1, 16)_ = 6.439** | **p < 0.05** | F _(1, 16)_ = 0.063 | p > 0.05 |
| **Amplitude of LE** | **F _(1, 16)_ = 6.644** | **p < 0.05** | F _(1, 16)_ = 2.122 | p > 0.05 |
| **Reversion by WAY-100635** | | | | |
| **RM two-way ANOVA** | **Treatment**  **F_(DFn, DFd)_** | **p value** | **Lesion**  **F_(DFn, DFd)_** | **p value** |
| **Latency of EE** | F_(1, 17)_ = 1.653 | p > 0.05 | F_(1, 17)_ = 4.388 | p > 0.05 |
| **Duration of EE** | **F_(1, 17)_ = 5.278** | **p < 0.05** | F_(1, 17)_ = 0.00163 | p > 0.05 |
| **Amplitude of EE** | F_(1, 17)_ = 1.934 | p > 0.05 | **F_(1, 17)_ = 4.923** | **p < 0.05** |
| **Latency of I** | F_(1, 12)_ = 0.2435 | p > 0.05 | F_(1, 12)_ = 0.0025 | p > 0.05 |
| **Duration of I** | F_(1, 12)_ = 1.079 | p > 0.05 | F_(1, 12)_ = 0.564 | p > 0.05 |
| **Latency of LE** | F_(1, 17)_ = 0.6076 | p > 0.05 | F_(1, 17)_ = 0.8752 | p > 0.05 |
| **Duration of LE** | **F_(1, 17)_ = 6.338** | **p < 0.05** | F_(1, 17)_ = 2.863 | p > 0.05 |
| **Amplitude of LE** | F_(1, 17)_ = 0.02247 | p > 0.05 | F_(1, 17)_ = 3.693 | p > 0.05 |

| **Effect of WAY-100635 on electrophysiological parameters in SNr neurons** | | | | |
| --- | --- | --- | --- | --- |
| **RM two-way ANOVA** | **Treatment**  **F_(DFn, DFd)_** | **p value** | **Lesion**  **F_(DFn, DFd)_** | **p value** |
| **Firing Rate** | F _(2, 22)_ = 0.1088 | p > 0.05 | F _(1, 11)_ = 0.0122 | p > 0.05 |
| **CV** | F _(2, 22)_ = 0.1627 | p > 0.05 | F _(1, 11)_ = 0.01374 | p > 0.05 |
| **Reversion by buspirone** | | | | |
| **RM two-way ANOVA** | **Treatment**  **F_(DFn, DFd)_** | **p value** | **Lesion**  **F_(DFn, DFd)_** | **p value** |
| **Firing Rate** | F _(2, 22)_ = 0.4748 | p > 0.05 | F _(1, 11)_ = 0.03117 | p > 0.05 |
| **CV** | F _(2, 22)_ = 0.3785 | p > 0.05 | F _(1, 11)_ = 0.09906 | p > 0.05 |
| **Effect of WAY-100635 on cortically evoked activity parameters in SNr neurons** | | | | |
| **RM two-way ANOVA** | **Treatment**  **F_(DFn, DFd)_** | **p value** | **Lesion**  **F_(DFn, DFd)_** | **p value** |
| **Latency of EE** | F_(2, 16)_ = 1.84 | p > 0.05 | F_(1, 8)_ = 0.6351 | p > 0.05 |
| **Duration of EE** | F_(2, 16)_ = 0.2351 | p > 0.05 | F_(1, 8)_ = 2.744 | p > 0.05 |
| **Amplitude of EE** | F_(2, 16)_ = 0.2054 | p > 0.05 | F_(1, 8)_ = 3.605 | p > 0.05 |
| **Latency of I** | F_(2, 20)_ = 0.4273 | p > 0.05 | F_(1, 10)_ = 0.0633 | p > 0.05 |
| **Duration of I** | F_(2, 20)_ = 1.434 | p > 0.05 | F_(1, 10)_ = 3.396 | p > 0.05 |
| **Latency of LE** | F_(2, 20)_ = 0.299 | p > 0.05 | F_(1, 10)_ = 4.388 | p > 0.05 |
| **Duration of LE** | F_(2, 20)_ = 2.658 | p > 0.05 | F_(1, 10)_ = 0.9979 | p > 0.05 |
| **Amplitude of LE** | **F_(2, 20)_ = 3.709** | **p < 0.05** | F_(1, 10)_ = 0.08574 | p > 0.05 |
| **Reversion by buspirone** | | | | |
| **RM two-way ANOVA** | **Treatment**  **F_(DFn, DFd)_** | **p value** | **Lesion**  **F_(DFn, DFd)_** | **p value** |
| **Latency of EE** | F_(2, 16)_ = 1.991 | p > 0.05 | F_(1, 8)_ = 1.37 | p > 0.05 |
| **Duration of EE** | F_(2, 16)_ = 0.2997 | p > 0.05 | F_(1, 8)_ = 0.7091 | p > 0.05 |
| **Amplitude of EE** | F_(2, 16)_ = 0.319 | p > 0.05 | F_(1, 8)_ = 0.1065 | p > 0.05 |
| **Latency of I** | F_(2, 20)_ = 3.225 | p > 0.05 | F_(1, 10)_ = 0.0878 | p > 0.05 |
| **Duration of I** | F_(2, 20)_ = 2.564 | p > 0.05 | **F_(1, 10)_ = 16.86** | **p < 0.05** |
| **Latency of LE** | F_(2, 20)_ = 1.406 | p > 0.05 | **F_(1, 10)_ = 8.432** | **p < 0.05** |
| **Duration of LE** | F_(2, 20)_ = 0.1975 | p > 0.05 | F_(1, 10)_ = 3.097 | p > 0.05 |
| **Amplitude of LE** | F_(2, 20)_ = 2.013 | p > 0.05 | F_(1, 10)_ = 0.6963 | p > 0.05 |
| **Effect of WAY-100635 on electrophysiological parameters in EP neurons** | | | | |
| **RM two-way ANOVA** | **Treatment**  **F_(DFn, DFd)_** | **p value** | **Lesion**  **F_(DFn, DFd)_** | **p value** |
| **Firing Rate** | F _(2, 22)_ = 0.9116 | p > 0.05 | F _(1, 11)_ = 0.2971 | p > 0.05 |
| **CV** | F _(2, 22)_ = 1.301 | p > 0.05 | F _(1, 11)_ = 0.4149 | p > 0.05 |
| **Reversion by buspirone** | | | | |
| **RM two-way ANOVA** | **Treatment**  **F_(DFn, DFd)_** | **p value** | **Lesion**  **F_(DFn, DFd)_** | **p value** |
| **Firing Rate** | F _(2, 22)_ = 0.4748 | p > 0.05 | F _(1, 11)_ = 0.03117 | p > 0.05 |
| **CV** | F _(2, 22)_ = 0.3785 | p > 0.05 | F _(1, 11)_ = 0.09906 | p > 0.05 |
| **Effect of WAY-100635 on cortically evoked activity parameters in EP neurons** | | | | |
| **RM two-way ANOVA** | **Treatment**  **F_(DFn, DFd)_** | **p value** | **Lesion**  **F_(DFn, DFd)_** | **p value** |
| **Latency of EE** | F_(2, 16)_ = 2.229 | p > 0.05 | F_(1, 8)_ 0.6319 | p > 0.05 |
| **Duration of EE** | F_(2, 16)_ = 0.9071 | p > 0.05 | F_(1, 8)_ = 0 | p > 0.05 |
| **Amplitude of EE** | F_(2, 16)_ = 0.2966 | p > 0.05 | F_(1, 8)_ = 0.1958 | p > 0.05 |
| **Latency of I** | F _(2, 20)_ = 0.4716 | p > 0.05 | F _(1, 10)_ = 0.0058 | p > 0.05 |
| **Duration of I** | F _(2, 20)_ = 2.39 | p > 0.05 | F _(1, 10)_ = 1.605 | p > 0.05 |
| **Latency of LE** | F_(2, 18)_ = 1.127 | p > 0.05 | F_(1, 9)_ = 0.2298 | p > 0.05 |
| **Duration of LE** | F_(2, 18)_ = 2.43 | p > 0.05 | F_(1, 9)_ = 0.504 | p > 0.05 |
| **Amplitude of LE** | F_(2, 18)_ = 0.3192 | p > 0.05 | F_(1, 9)_ = 0.1583 | p > 0.05 |
| **Reversion by buspirone** | | | | |
| **RM two-way ANOVA** | **Treatment**  **F_(DFn, DFd)_** | **p value** | **Lesion**  **F_(DFn, DFd)_** | **p value** |
| **Latency of EE** | F_(2, 16)_ = 3.555 | p > 0.05 | F_(1, 8)_ = 0.0081 | p > 0.05 |
| **Duration of EE** | F_(2, 16)_ = 0.2559 | p > 0.05 | F_(1, 8)_ = 0.1085 | p > 0.05 |
| **Amplitude of EE** | F_(2, 16)_ = 0.2365 | p > 0.05 | F_(1, 8)_ = 0.3023 | p > 0.05 |
| **Latency of I** | F_(2, 20)_ = 2.25 | p > 0.05 | F_(1, 10)_ = 0.3446 | p > 0.05 |
| **Duration of I** | F_(2, 20)_ = 0.1868 | p > 0.05 | F_(1, 10)_ = 2.821 | p > 0.05 |
| **Latency of LE** | F_(2, 18)_ = 0.3365 | p > 0.05 | F_(1, 9)_ = 0.2129 | p > 0.05 |
| **Duration of LE** | F_(2, 18)_ = 1.639 | p > 0.05 | F_(1, 9)_ = 0.0098 | p > 0.05 |
| **Amplitude of LE** | F_(2, 18)_ = 1,479 | p > 0.05 | F_(1, 9)_ = 0.08317 | p > 0.05 |

EE: Early Excitation; I: Inhibition; LE: Late Excitation.

**Supplementary Table 2.** **Effect of buspirone on firing properties of substantia nigra pars reticulata neurons in sham and 6-hydroxydopamine-lesioned rats.**

|  |  | **Basal** | **Buspirone** | | **WAY** |
| --- | --- | --- | --- | --- | --- |
|  |  |  | **0.6125 mg/kg** | **1.25**  **mg/kg** | **0.5 mg/kg** |
| **Sham (n = 14)** | **Firing rate (Hz)** | 27.4 ± 2.3 | 26.7 ± 2.8 | 27.0 ± 2.4 | 25.0 ± 2.6 |
|  | **CV (%)** | 30.8 ± 3.6 | 39.3 ± 5.6 | 39.1 ± 4.1 | 38.5 ± 4.7 |
|  | **Neurons exhibiting burst firing pattern (%)** | 40 | 60^&^ | 50 | 70^&^ |
|  | **Number of bursts** | 3.1 ± 1.8 | 6.5 ± 3 | 5.3 ± 3.2 | 3.9 ± 1.2 |
|  | **Duration of burst (ms)** | 0.8 ± 0.5 | 1.2 ± 0.75 | 1.4 ± 1.1 | 1.5 ± 1.1 |
|  | **Nº spikes/burst** | 23.4 ± 14.2 | 27.1 ± 14.7 | 49.3 ± 44.6 | 46.7 ± 28.7 |
|  | **Recurrence of burst (nº burst/min)** | 2.1 ± 1.2 | 4.3 ± 1.9 | 3.6 ± 2.2 | 2.9 ± 0.8 |
|  | **Intraburst frequency (spike/s)** | 31.6 ± 11.0 | 40.1 +10.9 | 41.2 ± 18.1 | 49.0 ± 11.6 |
| **6-OHDA (n = 13)** | **Firing (Hz)** | 27.6 ± 2.8 | 25.6 ± 3.6 | 25.0 ± 3.4 | 22.3 ± 2.6 |
|  | **CV (%)** | 57.9 ± 4.5* | 56.40 ± 6.7 | 57.35 ± 7.9 | 49.8 ± 7.5 |
|  | **Neurons exhibiting burst firing pattern (%)** | 76.9^#^ | 92.3^&^ | 81.8 | 75 |
|  | **Number of bursts** | 9.9 ± 4.1 | 11.9 ± 4.7 | 17.1 ± 6.3 | 12.7 ± 5.2 |
|  | **Duration of burst (ms)** | 0.5 ± 0.1 | 0.8 ± 0.3 | 0.6 ± 0.1 | 0.4 ± 0.1 |
|  | **Nº spikes/burst** | 14.9 ± 4.7 | 20.8 ± 6.1 | 13.9 ± 4.4 | 9.9 ± 2.4 |
|  | **Recurrence of burst (nº burst/min)** | 6.4 ± 2.6 | 9.3 ± 4.1 | 13.9 ± 4.4 | 10.6 ± 5.0 |
|  | **Intraburst frequency (spike/s)** | 41.5 ± 9.6 | 52.7 ± 9.7 | 41.0 ± 7.7 | 43.4 ± 10.3 |

Data are expressed as mean ± S.E.M. *p < 0.05 vs sham (RM two-way ANOVA followed by Bonferroni´s posthoc test), and Fisher´s exact test, ^&^p < 0.05 vs baseline and ^#^p < 0.05 vs sham.

**Supplementary Table 3.** **Effect of buspirone on firing properties of entopeduncular neurons in sham and 6-hydroxydopamine-lesioned rats.**

|  |  | **Basal** | **Buspirone** | **WAY** |
| --- | --- | --- | --- | --- |
|  |  |  | **0.6125 mg/kg** | **0.5 mg/kg** |
| **Sham (n = 13)** | **Firing rate (Hz)** | 20.39 ± 2.6 | 11.8 ± 2.2* | 19.9 ± 3.6 |
|  | **CV (%)** | 49.4 ± 7.7 | 64.9 ± 9.6 | 52.4 ± 9.9 |
|  | **Neurons exhibiting burst firing pattern (%)** | 75 | 66.7 | 63.6 |
|  | **Number of bursts** | 29.4 ± 13.7 | 10.1 ± 3.1 | 7.7 ± 2.9 |
|  | **Duration of burst (ms)** | 0.4 ± 0.1 | 0.7 ± 0.2 | 0.7 ± 0.3 |
|  | **Nº spikes/burst** | 12.6 ± 3.1 | 12.7 ± 3.2 | 7.6 ± 3.2 |
|  | **Recurrence of burst (nº burst/min)** | 19.7 ± 7.9 | 7.0 ± 1.9 | 4.9 ± 1.4 |
|  | **Intraburst frequency (spike/s)** | 45.1 ± 11.5 | 36.2 ± 10.1 | 27.2 ± 11.7 |
| **6-OHDA (n = 10)** | **Firing rate (Hz)** | 27.8 ± 3.4 | 24.9 ± 2.8 | 21.3 ± 3.5 |
|  | **CV (%)** | 56.3 ± 6.2 | 36.4 ± 4.4 | 47.5 ± 7.5 |
|  | **Neurons exhibiting burst firing pattern (%)** | 88.9^#^ | 55.6^&^ | 77.8^&^ |
|  | **Number of bursts** | 9.2 ± 5.4 | 3.8 ± 1.9 | 8 ± 4.2 |
|  | **Duration of burst (ms)** | 0.6 ± 0.3 | 0.2 ± 0.1 | 1.5 ± 1.2 |
|  | **Nº spikes/burst** | 25.6 ± 18.3 | 4.8 ± 1.8 | 9.7 ± 2.9 |
|  | **Recurrence of burst (nº burst/min)** | 5.1 ± 2.7 | 2.4 ± 1.1 | 5.9 ± 2.9 |
|  | **Intraburst frequency (spike/s)** | 55.9 ± 12.6 | 32.0 ± 10.8 | 35.9 ± 7.3 |

Data are expressed as mean ± S.E.M. *p < 0.05 vs baseline (RM two-way ANOVA followed by Bonferroni´s posthoc test), and ^&^p < 0.05 vs baseline and ^#^p < 0.05 vs sham (Fisher´s exact test).

**Supplementary Table 4.** **Effect of WAY-100635 on firing properties of substantia nigra pars reticulata neurons in sham and 6-hydroxydopamine-lesioned rats.**

|  | | **Basal** | **WAY-100635** | | **Buspirone** | |
| --- | --- | --- | --- | --- | --- | --- |
|  |  |  | **0.5 mg/kg** | **1 mg/kg** | **0.6125 mg/kg** | **1.25 mg/kg** |
| **Sham (n = 6)** | **Firing rate (Hz)** | 32.22 ± 5.44 | 30.96 ± 6.44 | 31.95 ± 8.6 | 33.2 ±7.6 | 30.47 ± 7.06 |
|  | **CV (%)** | 39.06 ± 11.27 | 32.41 ± 1037 | 34.50 ± 9.73 | 35.03 ± | 36.11 ± 9.73 |
|  | **Neurons exhibiting burst firing pattern (%)** | 50 | 33.33^&^ | 20^&^ | 60^@^ | 40^@^ |
|  | **Number of bursts** | 1.5 ± 0.718 | 0.83 ± 0.65 | 1.8 ± 1.8 | 2.6 ± 2.1 | 1.8 ± 1.56 |
|  | **Duration of burst (ms)** | 0.86 ± 0.433 | 1.15 ± 1.15 | 0.41 ± 0.41 | 0.44 ± 0.3 | 0.46 ± 0.4 |
|  | **Nº spikes/burst** | 30.31 ± 16.392 | 15.91 ± 15.2 | 4.6 ± 4.6 | 7.72 ± 4.8 | 6.72 ± 4.64 |
|  | **Recurrence of burst (nº burst/min)** | 1.11 ± 0.564 | 0.51 ± 0.4 | 1.33 ± 1.33 | 2.37 ± 1.96 | 1.44 ± 1.28 |
|  | **Intraburst frequency (spike/s)** | 18.6 ± 9.15 | 22.25 ± 19.8 | 2.1 ± 2.1 | 35.05 ± 19.08 | 10.5 ± 7.97 |
| **6-OHDA (n = 7)** | **Firing (Hz)** | 31.3 ± 5.6 | 34.30 ± 6.5 | 32.52 ± 6.5 | 30.79 ± 6.4 | 29.19 ± 6.36 |
|  | **CV (%)** | 33.9 ± 7.8 | 37.0 ± 14.52 | 41.07 ± 18.12 | 47.34 ± 21.45 | 48.35 ± 21.9 |
|  | **Neurons exhibiting burst firing pattern (%)** | 57.14 | 33.33^&^ | 50 | 50^@^ | 66.67 |
|  | **Number of bursts** | 4.14 ± 1.8 | 3.71 ± 3.8 | 9 ± 9.01 | 11.285 ± 11.09 | 9.42 ± 8.91 |
|  | **Duration of burst (ms)** | 0.415 ± 0.17 | 0.043 ± 0.03 | 0.788 ± 0.0214 | 0.09 ± 0.04 | 0.11 ± 0.05 |
|  | **Nº spikes/burst** | 17.99 ± 8.58 | 1.085 ± 0.76 | 32.76 ± 1.14 | 4.38 ± 2.36 | 5.8 ± 2.45 |
|  | **Recurrence of burst (nº burst/min)** | 2.79 ± 1.48 | 3.47 ± 3.6 | 6.35 ± 6.4 | 7.24 ± 7.09 | 7.46 ± 7.17 |
|  | **Intraburst frequency (spike/s)** | 33.91 ± 12.4 | 14.01 ± 10.7 | 34.59 ± 17.44 | 24.9 ± 14.58 | 37.29 ± 15.68 |

Data are expressed as mean ± S.E.M. ^&^p < 0.05, ^@^p < 0.05 vs WAY-100635 and ^#^p < 0.05 vs sham (Fisher´s exact test).

**Supplementary Table 5.** **Effect of WAY-100635 on firing properties of entopeduncular neurons in sham and 6-hydroxydopamine-lesioned rats.**

| **EP** | | **Basal** | **WAY-100635** | | **Buspirone** | |
| --- | --- | --- | --- | --- | --- | --- |
|  |  |  | **0.5 mg/kg** | **1 mg/kg** | **0.6125 mg/kg** | **1.25 mg/kg** |
| **Sham (n = 6)** | **Firing rate (Hz)** | 27.38 ± 6.48 | 27.07 ± 7.44 | 28.24 ± 6.92 | 28.13 ± 6.9 | 29.98 ± 7.3 |
|  | **CV (%)** | 35.27 ± 6.25 | 39.14 ± 6.55 | 32.64 ± 5.01 | 31.7 ± 5.37 | 31.66 ± 5.23 |
|  | **Neurons exhibiting burst firing pattern (%)** | 50 | 50 | 50 | 16.67^&@^ | 33.33^&@^ |
|  | **Number of bursts** | 0.7 ± 0.35 | 1.14 ± 0.553 | 0.428 ± 0.20 | 0.42 ± 0.42 | 2.85 ± 2.39 |
|  | **Duration of burst (ms)** | 0.9 ± 0.72 | 0.35 ± 0.242 | 0.083 ± 0.065 | 0.292 ± 0.292 | 0.36 ± 0.32 |
|  | **Nº spikes/burst** | 27.92 ± 24.13 | 10.4 ± 6.623 | 2.14 ± 1.38 | 3.42 ± 3.42 | 6.73 ± 5.49 |
|  | **Recurrence of burst (nº burst/min)** | 0.459 ± 0.23 | 0.78 ± 0.384 | 0.32 ± 0.15 | 0.284 ± 0.28 | 1.91 ± 1.58 |
|  | **Intraburst frequency (spike/s)** | 16.74 ± 9.64 | 19.025 ± 11.34 | 20.968 ± 14.035 | 1.683 ± 1.683 | 6.73 ± 4.628 |
| **6-OHDA (n = 7)** | **Firing (Hz)** | 28.14 ± 3.14 | 22.04 ± 5.5 | 20.08 ± 5.39 | 19.87 ± 5.39 | 20.25 ± 5.63 |
|  | **CV (%)** | 51.9 ± 16.8 | 46.49 ± 16.9 | 40.98 ± 11.63 | 44.30 ± 13.99 | 41.35 ± 11.16 |
|  | **Neurons exhibiting burst firing pattern (%)** | 57.14 | 42.86 | 57.14 | 42.86^#^ | 42.86 |
|  | **Number of bursts** | 18.7 ± 11.5 | 5.85 ± 3.8 | 4.7 ± 2.74 | 6.66 ± 4.69 | 6.57 ± 4.513 |
|  | **Duration of burst (ms)** | 0.13 ± 0.07 | 0.86 ± 0.7 | 0.44 ± 0.24 | 0.106 ± 0.068 | 0.14 ± 0.08 |
|  | **Nº spikes/burst** | 9.15 ± 5.72 | 12.8 ± 10.31 | 6.08 ± 2.53 | 2.52 ± 2.09 | 5.05 ± 3.088 |
|  | **Recurrence of burst (nº burst/min)** | 12.92 ± 8.11 | 4.08 ± 2.64 | 4.05 ± 2.62 | 1.74 ± 1.33 | 4.73 ± 3.36 |
|  | **Intraburst frequency (spike/s)** | 32.15 ± 16.01 | 13 ± 7.91 | 21.07 ± 11.06 | 9.93 ± 7.98 | 16.1 ± 8.66 |

Data are expressed as mean ± S.E.M. ^&^p < 0.05 and ^&^p < 0.05vs baseline, ^@^p < 0.05 vs WAY-100635 and ^#^p < 0.05 vs sham (Fisher´s exact test).

**
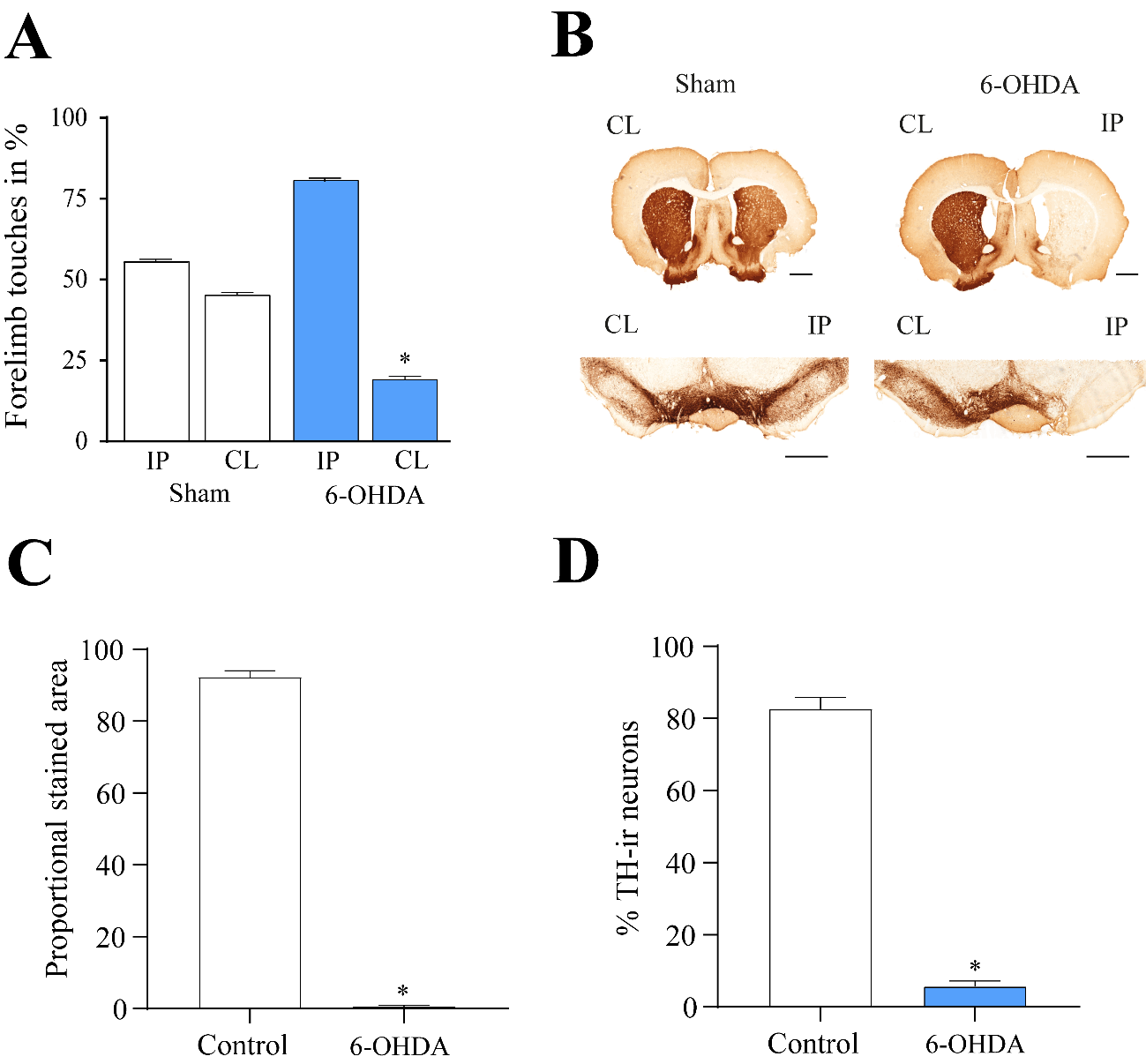
**

**Supplementary Figure 1. Validation of the 6-hydroxydopamine lesion.** **(A)** Motor asymmetry was evaluated comparing the use of the forelimb contralateral (CL) and ipsilateral (IL) to the lesion in the cylinder**. (B)** Coronal sections from sham and 6-OHDA group showing the lack of TH-immunoreactivity in the striatum and *substantia nigra*. Scale bar = 1 mm. (**C**) Histogram shows the proportional stained area of tyrosine hydroxylase (TH) in the striatum. (**D**) Histogram showing the density of TH-positive neurons in the substantia nigra obtained by stereology (5 – 6 sections per animal). Data is represented as mean ± S.E.M. *p < 0.05, two-tailed paired Student’s t test (n = 13 for sham group and n = 14 for 6-OHDA group).


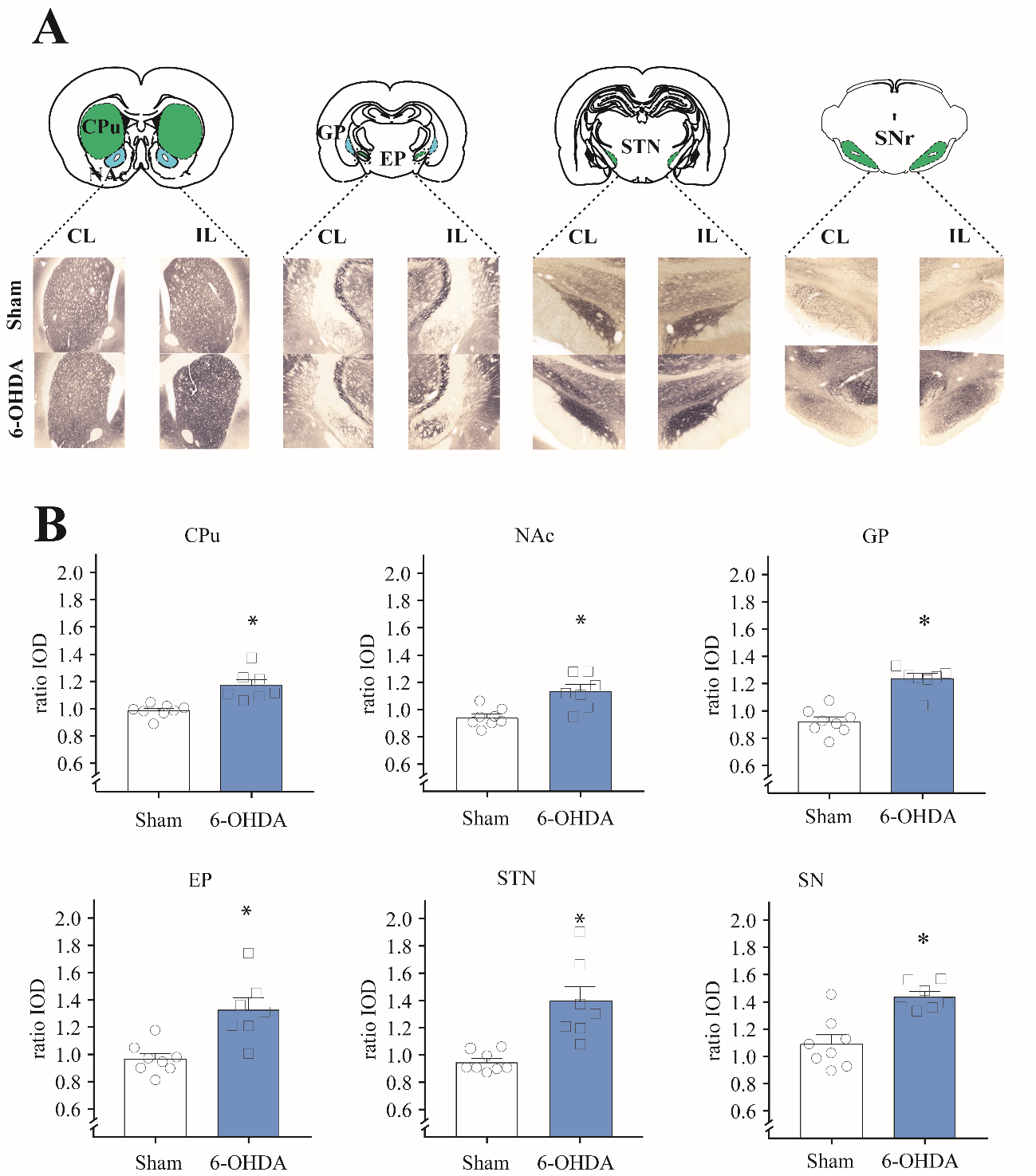


**Supplementary Figure 2. Cytochrome c oxidase reactivity in the basal ganglia nuclei. (A)** Representation of COX reactivity in the basal ganglia from sham and 6-OHDA lesioned rats. **(B)** The IOD of COX was expressed on the ratio between the ipsilateral (IL) and contralateral (CL) hemispheres in the two experimental groups. Different patterns were observed in the striatum (CPu), nucleus accumbens (NAc), external globus pallidus (GP), the entopeduncular nucleus (EP), the subthalamic nucleus (STN) and the *substantia nigra* (SN). The two experimental groups included in the analysis are sham (n = 7) and 6-OHDA-lesioned (n = 7). Each bar represents the mean ± S.E.M. of n rats. Each dot represents one subject. *p < 0.05 vs sham group (two‐tailed unpaired Student's t test).


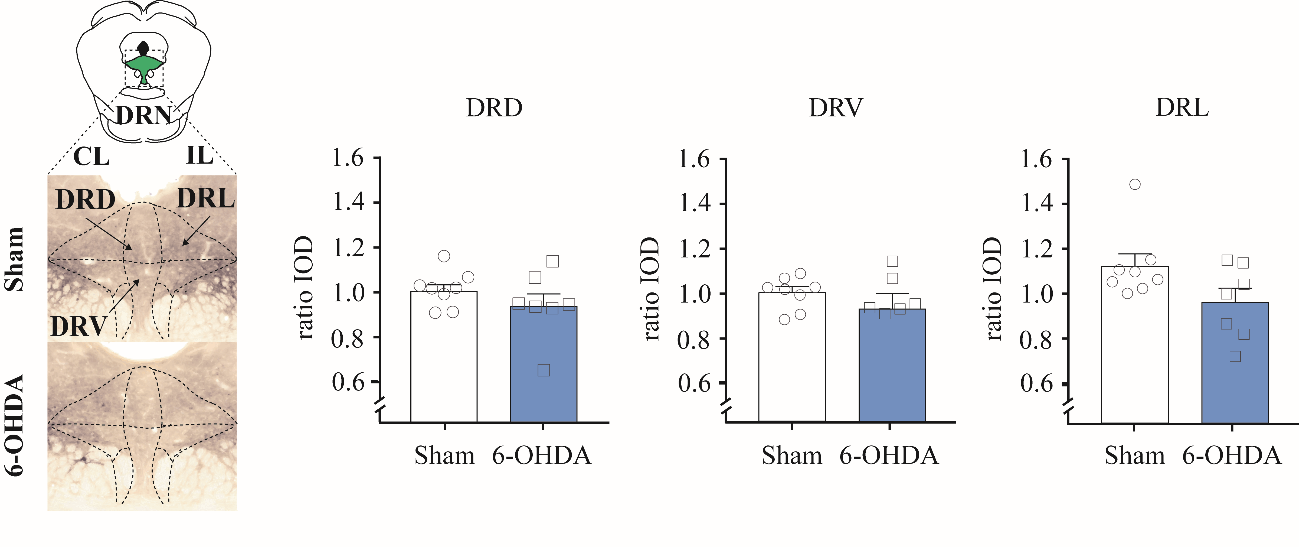


**Supplementary Figure 3. Cytochrome c oxidase reactivity in the dorsal raphe nucleus.** The IOD of COX was expressed on the ratio between the ipsilateral (IL) and contralateral (CL) hemispheres in the two experimental groups. Different patterns were observed in the dorsal (DRD), ventral (DRV) and lateral (DRL) regions. The two experimental groups included in the analysis are sham (n = 7) and 6-OHDA-lesioned (n = 7). Each bar represents the mean ± S.E.M. of n rats. Each dot represents one subject.
